# Predicting the Length of Stay of Cardiac Patients Based on Pre-Operative Variables—Bayesian Models vs. Machine Learning Models

**DOI:** 10.3390/healthcare12020249

**Published:** 2024-01-18

**Authors:** Ibrahim Abdurrab, Tariq Mahmood, Sana Sheikh, Saba Aijaz, Muhammad Kashif, Ahson Memon, Imran Ali, Ghazal Peerwani, Asad Pathan, Ahmad B. Alkhodre, Muhammad Shoaib Siddiqui

**Affiliations:** 1 Department of Computer Science, Institute of Business Administration, Karachi 75270, Pakistan; tmahmood@iba.edu.pk; 2 Department of Clinical Research Cardiology, Tabba Heart Institute, Karachi 75950, Pakistan; sana.sheikh@tabbaheart.org (S.S.); saba.aijaz@tabbaheart.org (S.A.); muhammad.kashif@tabbaheart.org (M.K.); ahson.memon@tabbaheart.org (A.M.); imran.ali@tabbaheart.org (I.A.); ghazalpeerwani43@gmail.com (G.P.); asadzpathan@gmail.com (A.P.); 3 Faculty of Computer and Information Systems, Islamic University of Madinah, Madinah 42351, Saudi Arabia; aalkhodre@iu.edu.sa (A.B.A.); shoaib@iu.edu.sa (M.S.S.)

**Keywords:** length of stay, Bayesian inference, hierarchical Bayesian regression, machine learning

## Abstract

Length of stay (LoS) prediction is deemed important for a medical institution’s operational and logistical efficiency. Sound estimates of a patient’s stay increase clinical preparedness and reduce aberrations. Various statistical methods and techniques are used to quantify and predict the LoS of a patient based on pre-operative clinical features. This study evaluates and compares the results of Bayesian (simple Bayesian regression and hierarchical Bayesian regression) models and machine learning (ML) regression models against multiple evaluation metrics for the problem of LoS prediction of cardiac patients admitted to Tabba Heart Institute, Karachi, Pakistan (THI) between 2015 and 2020. In addition, the study also presents the use of hierarchical Bayesian regression to account for data variability and skewness without homogenizing the data (by removing outliers). LoS estimates from the hierarchical Bayesian regression model resulted in a root mean squared error (RMSE) and mean absolute error (MAE) of 1.49 and 1.16, respectively. Simple Bayesian regression (without hierarchy) achieved an RMSE and MAE of 3.36 and 2.05, respectively. The average RMSE and MAE of ML models remained at 3.36 and 1.98, respectively.

## 1. Introduction

Length of stay (LoS) is essential for analyzing a patient’s severity, clinicians’ prognosis, institutional resources, and personnel allocation [1]. Efficient monitoring and estimation of this indicator lead to better financial and medical decisions for the staff and the patient. Given the global shortage of medical resources, health service providers rely heavily on LoS estimates to monitor patient influx and optimize waiting times. This is especially true for medical institutions in developing countries with high demand and scarce resources [2]. These factors are the driving force behind an increased interest in this area.

In the case of Pakistan, the problem of LoS prediction is even more significant for cardiac patients due to the high incidence of cardiovascular diseases and associated risks. According to [3], the estimated age-standardized cardiovascular disease incidence in Pakistan was 918.18 per 100,000 (global 684.33) in 2019. The age-standardized death rate was 357.88 per 100,000 (global 239.85). Given the worsening living conditions, this incidence is expected to increase further going forward. On top of it, specialized resources and professionals are scarce in Pakistan, which makes the problem of LoS prediction for cardiac patients even more paramount for resource optimization and personnel allocation.

Various methods, including subjective point estimates, machine learning algorithms, and regression analysis, have been used to forecast LoS. However, the healthcare data are generally high-dimensional, which warrants using more sophisticated methods instead of subjective estimates. Usually, robust statistical methods or machine learning algorithms are used to identify patterns and interactions among variables. When applied to healthcare data, these methods result in valuable insights and precise forecasts that facilitate decision-making [4]. Recently, deep learning methodologies are also being increasingly used in the healthcare industry. For example, refs. [5,6] have used artificial neural networks along with other machine learning techniques to identify morbidity and mortality associated with cardiac patients. However, the application of Bayesian inference methods has been limited, especially for LoS prediction in a cohort of cardiac patients from a developing country.

The distribution of data is difficult to approximate when the target variable is highly skewed and takes on values across a large domain. This variability in the target variable is often not captured by the predictive models and results in biased estimates and inefficient inference. Moreover, high variability often implies that the data contain outliers (extreme values), which adversely affect the performance of the models. Usually, the outliers are removed or constrained to a maximum value to smooth the variables. However, this results in losing valuable information about the causal interactions between the predictors and the target variable. Especially in the case of LoS, predictions on the data without outliers would hinder a clinician’s ability to identify the effect of variables that result in an extremely high or exceptionally low LoS. Another way to rectify the issue of high variability is to increase the sample size, with the assumption that some regularity will be induced in the target variable with an increased number of observations. However, it is not always feasible to collect more data, especially in the healthcare domain, where data security and sensitivity are paramount.

The target variable in this study (LoS) exhibits high variability (mean: 8.3, sd: 3.7, min: 1, max: 65), which makes it difficult to model the relationship between the response and the predictors. This type of (skewed) target distribution (similar to this study) is extensive in the literature [7,8,9]. Considering the nature of the dataset, Bayesian regression models are used and evaluated against various machine learning models to assess the regression accuracy and parameter interpretability. The aim is to facilitate and aid the healthcare community in exploring the Bayesian paradigm for modeling patient LoS. The study also evaluates key differences between a simple Bayesian regression model and a hierarchical model by creating ‘soft divisions’ of the dataset. The results show that by reframing the problem in a hierarchical paradigm, a better explanation of the changing effects of the predictors on the target variable can be obtained. Furthermore, the results show that the hierarchical approach successfully captures the variability of the target variable by approximating a posterior that is close to the actual distribution, which leads to better predictions and sound causal analysis.

This retrospective cross-sectional study evaluates the use of Bayesian regression models (simple and hierarchical) for LoS prediction for cardiac patients (based on their pre-operative clinical features only) who had undergone cardiac bypass surgery (CABG) at Tabba Heart Institute, Karachi, Pakistan, between 2015 and 2020. An empirical comparison is drawn between the results of Bayesian and frequentist models such as support vector regression (SVR), multiple linear regression (MLR), Huber regression (HR), lasso regression (LR), ridge regression (RR), random forest regression (RF), extreme gradient boosting regression (XGBR), and stochastic gradient descent regression (SGRR). The best-performing model is then used for the interpretability of the parameter estimates along with their causality. The study uses the target variable of LoS and tries to model the response in terms of its predictors to present an application of Bayesian regression models that could handle outliers without removing them or adding more data.

The rest of the study is organized as follows: Section 2 explores related work; Section 3 explains the methodology in detail; Section 4 discusses results; and Section 5 concludes the study.

## 2. Related Work

With the introduction of electronic health record (EHR) systems in healthcare institutions, a new research paradigm of statistical, machine, and deep learning methodologies has emerged. Many researchers have used the clinical data of patients for disease inference, clinical deterioration, decision support systems, optimization, and outcome prediction [10,11]. One of the most prevalent research areas is length of stay prediction. Due to its vast implications and the absence of a universal benchmark framework, clinicians, health professionals, and even the machine and deep learning communities have shown increasing interest in the domain.

For example, Colella et al. use a machine learning classifier to evaluate the delaying effects of lower limb fractures on LoS [12]. Instead of using regression models, the authors divided the target variable into two distinct bins of continuous values. A machine learning classifier then predicted the LoS class for each inpatient. Similarly, another study by Colella et al. evaluated the accuracy of multiple machine learning models for LoS prediction for pediatric patients [13]. The researchers used random forest, naïve Bayes, support vector machines, and logistic regression to classify the patients into their LoS categories. Reimagining the problem (of LoS prediction) as a regression problem (instead of a classification one), a team of researchers from San Giovanni di Dio e Ruggi d’Aragona’ University Hospital (Italy) used multiple regression models to predict LoS for the patients undergoing femur fracture surgery [14]. Another team of researchers used machine learning algorithms to predict patients’ duration of surgery (DoS) and LoS [15]. They used the data of patients undergoing total knee arthroplasty queried from the American College of Surgeons (ACS) National Surgical Quality Improvement (NSQIP) database. The results of the study showed improved predictive performance with Pytorch multi-layer perceptrons (MLPs) on testing and validation datasets. A study by Barsasella et al. evaluated the applications of machine learning algorithms in predicting LoS and mortality for patients with type 2 diabetes mellitus and hypertension. They used insurance claim data from the Dr. Soekardjo Regional Public Hospital, Indonesia. The results of the study showed superior performance of MLPs and linear regression for the problems of mortality and LoS prediction, respectively [16]. Another team of researchers from Tianjin Medical University General Hospital, China, analyzed the applications of machine learning models to the LoS of patients with femoral neck fractures. The study used artificial natural networks with one hidden layer along with SVR and a principal component regression (PCR) model for estimations. The study reports superior estimates with the PCR model, with a mean absolute error of 1.525 [17].

A group of researchers at the Academic and Educational Hospital of Rajaei Cardiovascular Medical & Research Center in Tehran [18] evaluated the importance of LoS prediction in high-risk cardiac patients in Iran and used decision trees, support vector machines (SVMs), and artificial neural networks (ANNs) for the classification of patients into their predicted LoS class. Working in the same paradigm, Wright et al. used statistical methods to evaluate factors that resulted in prolonged LoS in heart failure patients admitted to Auckland Hospital (New Zealand) [19]. A team of researchers from St. James Hospital (Ireland) devised a deep learning framework using pre- and post-operative variables to stratify LoS in cardiac patients with high morbidity and readmission risk [20]. In another study, Jack et al. used neural networks to predict LoS for cardiac patients admitted to the ICU of St. Michael’s Hospital, Toronto, Canada [21]. Morton et al. used multiple machine learning methods (SVM, decision trees, and multiple linear regression) to predict long- and short-term LoS in diabetic patients [22]. A similar study by Chuang et al. assessed the post-surgical, prolonged LoS of ICU patients at a teaching hospital in Taiwan using various supervised learning algorithms (decision trees, support vector machines, and random forests) [23].

Omachonu et al. conducted a study on patients at a teaching hospital in the USA, where they estimated the LoS of the inpatients using regression methods [24]. In a similar study, Khosravizadeh et al. evaluated the factors affecting LoS in patients of a teaching hospital in Iran [25]. According to the researchers, age, employment, marital status, history of previous admission, patient condition at discharge, methods of payment, and type of treatment influenced the LoS. Mekhaldi et al. used random forests and gradient-boosting models to predict LoS [26]. A real-time predictive framework capable of predicting mortality, readmission, and LoS was devised by [27]. They used the electronic medical records (EMR/EHR) of the patients admitted to Sydney Metropolitan Hospital (from 2008 to 2013) to fine-tune their models in real time with the addition of the new data. This online predictive model proved more accurate than offline models due to continuous retraining and evaluation. A study by Li et al. on a Chinese cohort identified days before the operation, wound grade, operation approach, charge type, and the number of admissions as key factors in predicting LoS [28]. The researchers used a neural network algorithm with backpropagation to achieve an accuracy of 80%. Unlike most studies on LoS predictions (using frequentist methods), Saez-Castillo et al. evaluated Bayesian techniques to evaluate the factors leading to nosocomial infections and the resulting prolonged LoS in patients at a Spanish hospital [29]. Another study by Angus et al. evaluated the factors affecting prolonged LoS in hospitals. The authors used hierarchical mixture regression to model the maximum likelihood for the heterogeneously distributed LoS of inpatients [30]. Another study by Tang et al. used MCMC methods for a LoS classification model [31]. To account for the variability in the target variable, the researchers used the Coxian-Phase type regression method.

The literature on LoS prediction is laden with machine and deep learning methodologies, but there are a few studies evaluating Bayesian inference methods. Research studies by Saez, Angus, and Tang et al. explored the Bayesian paradigm in sufficient detail for LoS prediction. The authors employed various methods to account for variance in the target variable. Saez-Castillo et al. used Bayesian networks to learn the latent relationships between the predictors. These hidden relationships among the variables are useful to account for variance in the data. However, there is still a need for a comparative analysis of Bayesian and ML regression models for LoS prediction on highly skewed target variables. Additionally, identifying causality among predictors and the target variable of LoS, along with their varying effects subject to the hierarchy, often remains unstudied. This study examines Bayesian models (especially the hierarchical regression model) against various ML models to draw predictive comparisons, which are then used as the basis for causal analysis to identify key factors that lead to higher or lower LoS (and, in turn, morbidity) in cardiac patients.

## 3. Methods

The data of adult male and female patients undergoing CABG at Tabba Heart Institute (THI) between 2015 and 2020 were queried from the in-house cardiothoracic surgery registry (curated following the model of the Society of Cardio-Thoracic Surgery (STS) database) maintained at the hospital. The data is extracted starting in 2015, after the STS database was fully implemented at THI with little missing data. The inclusion criteria are based on individuals (male and female) admitted at THI between 2015 and 2020 for either elective, urgent, emergent, or emergent salvage CABG procedures. Only pre-operative features collected by medical staff before surgery are used for the study due to the relative importance of the early prediction of LoS. The pre-operative, static variables were used as exogenous variables, with LoS as the target variable. The dataset contains 5363 observations and 68 variables, including the target variable of LoS.

**Figure 1:**
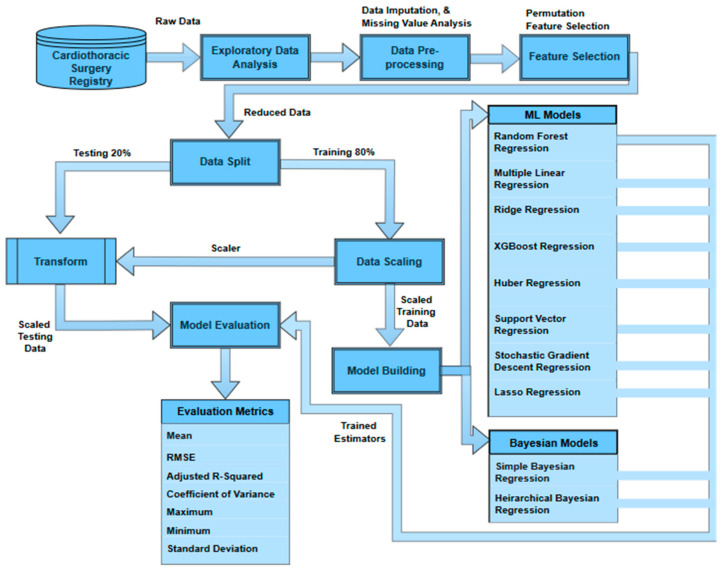
Overview of the methodology.

The Figure 1 shows the overview of the methodology used in this study. The data is first extracted from the THI database and then is explored and audited to understand the patterns, trends, and inconsistencies. The data imputation is performed to cater to the missing values and prepare the data for the feature selection process using the permutation feature importance (PFI) method. The reduced dataset is then used to split the data into training sets (80%) and testing sets (20%). The training data are then scaled to a range of 0–1 using min–max scaling to minimize the model bias due to the relative magnitudes of the features. The scaler used to fit the training data is used to transform the testing set to ensure no data leakage. The training set is divided again into the same ratio of 80–20 to train a base model (random forest regressor) on the 80% training set. The remaining 20% of the training set is then used to recursively remove a feature and test its importance. Later, Bayesian and ML models are trained and tested on the reduced feature space. The results are then evaluated against multiple metrics (root mean squared error, mean absolute error, mean, standard deviation, minimum, maximum, coefficient of variance, and adjusted R-squared) to find the best estimator.

### 3.1. Data Characteristics

**Figure 2:**
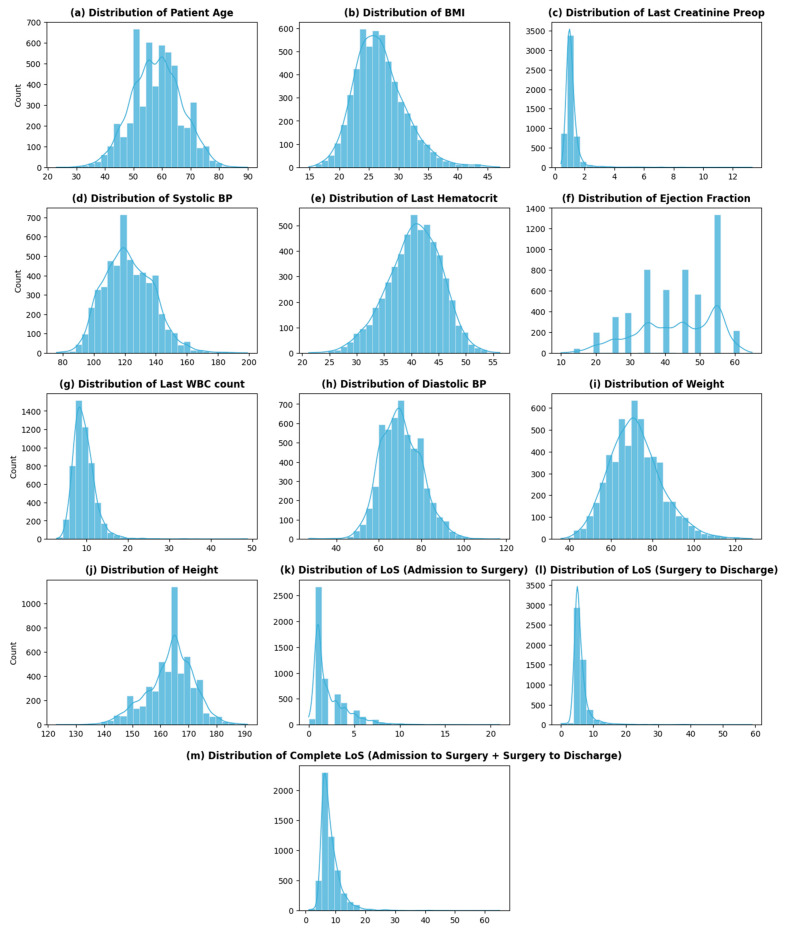
Distribution of continuous variables.

Table 1a,b shows an overview of a few dataset variables along with their percentage of missing values (Appendix A: Table A1 contains a complete list of dataset variables, their descriptions, their characteristics, and the percentage of missing values). Among the 68 variables, 12 are continuous with varying distributions (Figure 2), while 52 are categorical variables. The remaining variables are datetime (3) and an identifier (1) for each distinct patient.

The dataset contains two variables that impart information about patients’ stay at THI before and after CABG: *admission_to_surgey* (number of days it takes per patient to go into CABG surgery once admitted) and *LOS_surgery_to_discharge* (number of days it takes per patient to be discharged after the surgery—recovery period). The overall LoS of a cardiac patient at THI is then the sum of these two variables. The distribution of the overall LoS of patients at THI is shown in Figure 2m. As seen from the graph, the distribution of the LoS is skewed, with the highest frequency at 6 (days) and an average LoS of around 8 (days). The distribution of LoS shows high variability (sd—3.6), with some large values of LoS (>20 days) present at the extreme.

**Figure 3:**
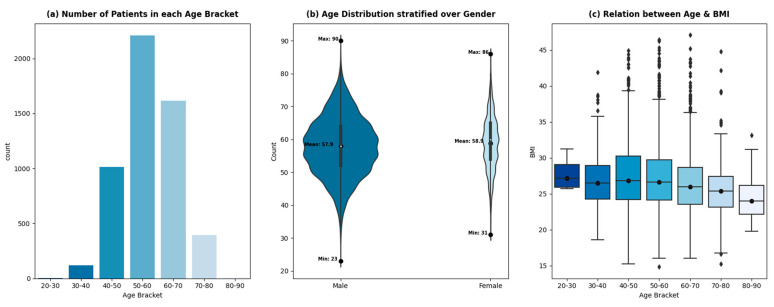
Relationship between age, gender, and BMI.

The variable of age is divided into equal ranges (of 10 years) to analyze the trend of patient admissions with age. Figure 3a shows a sharp drop in patients after 70 years, which signifies a low proportion of patients being admitted for CABG procedures with an age greater than or equal to 70 years. This phenomenon can be attributed to multiple factors. Firstly, the incidence of mortality due to cardiovascular diseases is higher in elderly patients as compared to non-elderly patients [32,33]. This should ideally result in a higher influx of patients with an age greater than 70 being admitted for a treatment procedure. However, treating elderly patients for the adverse effects of cardiovascular diseases poses perioperative complications, due to which healthcare professionals treat the symptoms rather than the disease’s cause.

With the advent of new, sophisticated procedures, the overall incidence of complications in elderly patients has steadily declined [34]. However, it will take time for it to reflect in the data due to the slow relative adoption in developing countries. The other factor contributing to the low population of elderly patients admitted at THI for CABG is the low life expectancy in Pakistan (65 years) [35].

The ages of the patients being admitted for CABG procedures at THI from 2015–2020 are distributed normally, with an average age of 58 years. The age distribution of the patients stratified by gender is shown in Figure 3b. The female age distribution shows regular spikes at 50, 55, and 60 years, whereas the age distribution of male patients shows a smoother trend. The average age of male and female patients remains comparable (57.9 and 58.9, respectively). The relative width of the plots shows the gender disparity within the dataset. Around 18% of the patients admitted for CABG are female, while 82% are male. A total of 63% of the patients are classified as overweight with a BMI greater than 25 kg/m^2^, which increases the risk of cardiovascular diseases, according to [36,37]. The prevalence of being overweight exists across all age brackets, however, with different proportions—Figure 3c.

**Figure 4:**
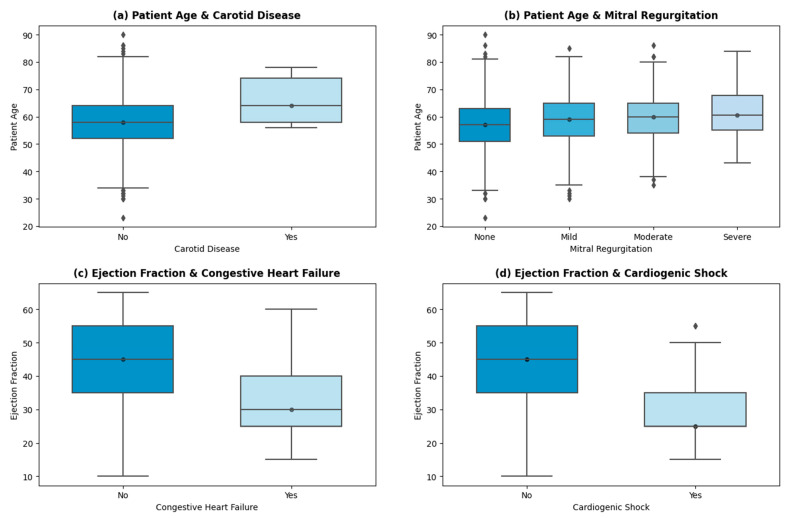
Important insights.

Further analysis of the cohort uncovered a few insights shown in the graphs in Figure 4.

A linear trend is shown (in Figure 4a) between the patient’s age and the incidence of carotid artery disease (CD). The average age of patients suffering from CD is 66 years, while the average age of patients with no indication of CD is 58 years. Advancing age results in arterial stiffness, structural deformation, and other age-inducing functional alterations, which correlate with a higher risk of carotid disease [38,39,40]. Similar trends are persistent in other high-risk factors, such as arrhythmia and cardiogenic shock. THI uses echocardiography as the primary test for mitral insufficiency/regurgitation. The stratification of the patients into *None*, *Mild*, *Moderate*, and *Severe* categories is then based on American Society of Echo (ASE) guidelines [41]. Severe mitral regurgitation (MR) is seen in patients with higher ages (with an average age of 61 years). The age of patients with mild to moderate levels of MR is 59 years on average, while patients with no signs of MR have an average age of 56 years. Other valvular regurgitation diseases (aortic and tricuspid) also showed a positive linear trend with age, which is endorsed by ref. [42].

The incidence of congestive heart failure (HF) is higher in patients with a mean left valve ejection fraction (EF) of 32%. A total of 329 patients showed HF with reduced EF (less than 40%), 75 showed HF with mid-range EF (between 40% and 49%), and 52 encountered HF with preserved EF (levels greater than 50%). The patient stratification of HF at THI concerning EF levels is based on US guidelines [43,44]. As per Figure 4d, patients with cardiogenic shock presentation on admission have a mean EF of 29.6%. Cardiogenic shock is caused by decreased contractility or decreased filling of the heart, mostly the former. The low contractility causes low cardiac output and depressed ventricular systolic function, which is persistent in patients with reduced HF [45].

#### 3.1.1. Data Cleaning and Imputation

The analysis and audit of the data showed a very minimal amount of missing data (≈2.53% cumulative). The variable *BPsystolic* shows the highest missing rate of 0.48%, followed by *diastolic*, *pulmonary_artery_done*, *last_wbc_count*, *BMI*, and *pulmonary_insufficiency* with identical missing rates of 0.41%.

The variables were imputed with other variables as a reference. For example, the data for *BMI* was filled with calculated *BMI* using *weight* and *height* parameters. Similarly, the variable *BPsystolic* was imputed by the dichotomous variable of *hypertension*. The values of *BPsystolic* were averaged against patients whose hypertension label was ‘*Yes*’ (mean—124 mmHg). Similarly, for patients with hypertension labeled ‘*No*’, a different mean of *BPsystolic* was acquired (mean—117 mmHg). These two mean values were then used to fill in the missing values of the variable *BPsystolic*, with hypertension as a reference. A similar strategy was used to impute values for the diastolic variable as well. The observations with missing values for *pulmonary_artey_done*, *last_wbc_count*, and *pulmonary_insufficiency* attributes account for 1.3% of all the missing values. The cumulative missing rate for these variables is less than the benchmark of 5% and hence can be safely removed [46] without any adverse effects on the model predictions.

#### 3.1.2. Data Preparation

The variable *TempSNO* is removed from the model-building process as it is an identifier for a patient and does not contribute to the estimation. Similarly, all datetime variables (*date_of_discharge*, *date_of_admission*, and *date_of_surgery*) were also removed from the dataset as the information imparted from these variables is already encoded in the variables *Admission_to_surgery* (days) and *LOS_Surgery_to_discharge* (days). The variables *weight* and *height* were subsequently removed after calculating and filling in the *BMI* variable.

To estimate the overall LoS of a cardiac patient at THI, the variables *Admission_to_surgery* and *LOS_Surgery_to_discharge* are added together to create a new target variable, *LOS*. Consequently, these variables are removed from the dataset after creating the new target variable. The resulting dataset has 61 features (including the newly created target variable LoS).

All the categorical variables are label encoded (starting with 0), and the continuous variables are scaled between 0 and 1 (Min–Max scaling). Min–Max scaling retains the natural order (distance) between the data points while reducing the effect of their magnitude on the target variable. Without this normalization, variables with large values can result in biased estimates. The normalization step is performed after splitting the dataset into training and testing sets. The scaler is fitted to the training data and then used to transform both the training and testing datasets to minimize data leakage. The scaling parameters learned on the training set are used to transform the testing set as well, because scaling parameters for testing data are not known in real life. Hence, using the scaling parameters of the testing set for the scaling of testing data would make the model aware of the distribution of the testing set, introducing leakage and overestimated accuracy.

### 3.2. Permutation Feature Importance (PFI)

PFI evaluates the increase in the models’ score when a feature effect is removed. The change in model accuracy before and after the removal of the variable gives an insight into the relative importance of a feature [47]. PFI is used due to its robustness against overfitting. If the dataset contains uncorrelated variables, then a feature selection method can increase the effect of a feature that is not predictive of the outcome [48]. The PFI method solves this issue by testing the variable’s importance in terms of model accuracy. It ensures that only those features are included in the sample space that contribute positively to the accuracy of the model. In addition to gauging the variable’s importance, PFI helps to gauge the feature interactions and their contribution to the loss as well. It perturbs the feature of interest, effectively removing its interaction with other variables. The relative change in the model performance is then indicative of main and interaction feature effects. Impurity-based feature selection methods (decision trees and random forests) show a bias towards high cardinal and numerical variables as they show greater affinity towards the scale of measurement of a variable [49]. The dataset of this study contains numerical variables with different magnitudes and categorical variables with high cardinality, which results in under- or overestimated variable influence using impurity-based feature selection methods. Greedy algorithms for feature selection, such as recursive feature elimination (RFE) and sequential feature selection (SFS), are good at removing redundant variables from a dataset, resulting in an optimum number of features defined *a priori*. These methods recursively add or remove a feature from the dataset, retrain the model, and gauge the changes in model performance, which makes them undesirable for datasets with a large number of variables due to increased runtimes. Furthermore, defining a suitable number of reduced parameter spaces *a priori* is suboptimal for the overall performance of the model. Defining a low number of features would result in an underperforming model, and a large number would result in high time complexity. This study aims to predict the LoS for cardiac patients where a tradeoff on accuracy is not desirable. Hence, PFI is used as a feature selection method to ensure the presence of only those variables that positively contribute to the accuracy of the model.

PFI requires a trained model (on all features) with known accuracy. The variables are then removed recursively to assess the change in accuracy. This research uses a random forest regressor (trained on 60 independent variables) as the base model to assess feature importance.

To assess the effect of a variable j on the model’s overall performance, the effect of column j must be removed and replaced by a vector of the same dimension. This new vector, or augmented feature vector, is sampled from the same distribution as the feature of interest. This ensures a break between the target and the feature, hence simulating the effect of the absence of the feature. The model is tested on augmented data (with the permutated feature), and the new score is determined. The change in the score translates into the importance of the said feature.

Let $j_{i}$ be a feature of the dataset *D* such that each $j_{i}$ belongs to the feature set J, where $J=\{j,\;j_{2},j_{3},\ldots ,j_{n}\}$. If the output of the model trained on dataset *D* is $\hat{y}$, then the output of the model on augmented dataset $D_{J\backslash \left\{j_{i}\right\}}$ (dataset with $j_{i}$ permutated column) is the average accuracy over K permutations of feature $j_{i}$, given as $\frac{1}{K}\sum_{1}^{K}{\hat{y}}_{k,J\backslash \left\{j_{i}\right\}}$. The change in accuracy or permutation feature importance of $j_{i}$ is then given by:

(1) $$s_{j}=\hat{y}-\frac{1}{K}\sum_{1}^{K}{\hat{y}}_{k,J\backslash \left\{j_{i}\right\}}$$

The training dataset is divided into two further sets of training and testing samples. A total of 80% of the training data are used to train a Random Forest regressor with the complete feature set (60 variables). The remaining 20% of the training data are then used to recursively test the model’s performance when a feature effect is removed. One way to remove the feature effect is to remove that feature from the feature set entirely. However, as the baseline model is trained on all independent variables (60 variables), the model expects 60 variables for testing as well. Hence, removing a feature would result in an error while testing the model’s performance. That is why a vector of the same dimension (as the feature to be removed) is sampled from the same distribution as that of the variable of interest. For instance, to measure the importance of *last_cretenine_preop* in predicting the LoS, the variable is replaced with the randomized version of the same feature in the test set. This randomization removes any information or effect that *last_cretenine_preop* may have on the target variable. The model is then tested on the validation set, and the resultant RMSE is compared to the RMSE with all the features. Any increase or decrease in the resultant RMSE will dictate the effect of *last_cretenine_preop* on the LoS.

Table 2 shows the features selected by the permutation feature selection method with a decreasing order of change to the accuracy of the model.

### 3.3. Models

#### 3.3.1. Simple Bayesian Regression Model (SBM)

To model the problem of LoS prediction in the Bayesian paradigm, it is imperative that an empirical relationship be identified that links the target variable with its parameters. Consider the following relationship:

(2) Log(T)=Xβ+ϵ

The above equation is similar to an accelerated failure time (AFT) model proposed by ref. [50], which is used to predict the occurrence of an event in time *T*. If the event in the AFT model is taken as the influx of patients, then the time *T* can be thought of as the expected length of stay for a patient. Rewriting the above equation tailored to the LoS problem gives:

(3) $$Log(Y)=\beta_{0}{+X}^{\prime }\beta^{\prime }+\epsilon$$

*Β*′ is a vector of coefficients of dimension n, where *n* = 44 is the number of features determined by the permutation feature selection method. Each *β* is sampled from a prior distribution. A prior distribution is knowledge about the dataset’s variables before any evidence is considered. It can be any probability distribution based on domain knowledge and variable characteristics. *X*′ is the training data of dimensions *m* × *n*, where *n* is the reduced feature space, *m* is the number of training samples (80% of the data), and *β*_0_ is the intercept.

Given the target variable’s continuous and numerically positive nature, posterior sampling must be conducted from an always positive continuous probability distribution. A truncated normal distribution is used as the likelihood for SBM. The truncation is performed to keep the values continuous and positive. The priors for the model can be initiated with weakly informative priors [51]. The model specifications for SBM are then:

(4) $$\beta^{\prime }\sim Normal\left(\mu_{\beta^{\prime }},\sigma_{\beta^{\prime }}\right),n=44\;\beta_{0}\sim Normal\left(\mu_{\beta_{0}},\sigma_{\beta_{0}}\right),n=1\;\epsilon \sim Normal\left(\mu_{\epsilon },\sigma_{\epsilon }\right)$$

where $\mu_{\beta^{\prime }}$, $\mu_{\beta_{0}}$, $\sigma_{\beta^{\prime }}$, and $\sigma_{\beta_{0}}$ are the hyperpriors and are sampled from normal and half-normal distributions, respectively.

For a simple Bayesian regression model (SBM), the equation for likelihood is:

(5) $$\;P\left(y|X^{\prime };\beta^{\prime },\beta_{0}\right)=\prod_{i=1}^{n}{\frac{1}{\sqrt{2\pi }\sigma }e}^{-\frac{1}{2}{\left(\frac{y_{i}-\beta^{\prime }\cdot {X^{\prime }}_{i}+\beta_{0})}{\sigma }\right)}^{2}}$$

The Markov Chain Monte Carlo (MCMC) simulation method [52] is used to estimate the posterior resulting from the above priors and the likelihood function. The MCMC method iteratively samples values from the posterior until convergence or equilibrium is reached (the transition probability reaches the stationary probability distribution). The parameters that result in the said convergence are the estimated parameters of the model. The sampling process is governed by a sampling algorithm such as the Metropolis algorithm [53], Gibbs sampler [54], and No-U-Turn Sampler (NUTS) [55].

To approximate the posterior distribution resulting from the above-mentioned model specification, the study uses the implementation of a No-U-Turn Sampler (NUTS) from the PyMC package [56].

#### 3.3.2. Hierarchical Bayesian Regression Model

This study evaluates hierarchical Bayesian regression in addition to simple Bayesian regression, as it is more robust to the variability of the data. Most of the values of LoS are concentrated between the ranges of 0–10, with some values spread far apart. Building a non-hierarchical Bayesian regression model would then result in a bias towards values with a high frequency and would not be able to predict tail values. One way to improve model performance is to divide the dataset into smaller parts such that each subset is locally similar with low variability. Smaller models are trained on each subset of the data. The combination of these smaller models is known as an un-pooled model and can result in better accuracy. However, this kind of model is prone to overfitting due to small subsets of the data. All the models are independent of each other, with no knowledge transfer in between. Latent information is lost and results in deficient performance on test sets. On the contrary, multilevel, hierarchical, or partially pooled models allow knowledge sharing between sub-models, which helps them capture the variability of the target variable.

**Figure 5:**
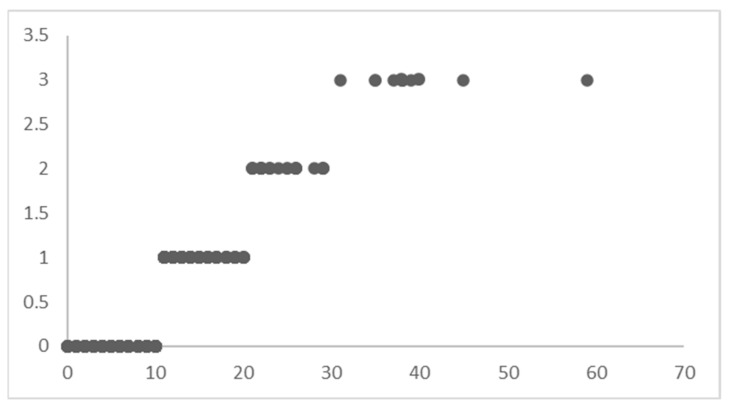
Levels of LoS.

To use the method of hierarchical Bayesian regression, the target variable of LoS is divided into four distinct ranges of values (Figure 5).

The four LoS levels do not mean that the dataset is divided into subsets (as in an un-pooled model); instead, it is indexed to let the model know that it must learn different posterior distributions for these levels of LoS yet maintain the optimum flow of knowledge between the distributions to capture the variability in the best possible way.

The records of patients with an LoS between 0–10 are indexed with 0, the LoS between 10–20 are indexed with 1, the LoS between 20–30 are indexed with 2, and the LoS greater than 30 are indexed with 3. This indexing of the dataset based on the LoS ranges acts as ‘levels’ for Bayesian regression. The priors are initialized differently for each ‘level’ in the dataset. Instead of sampling 44 priors for *β*′, 1 for *β*_0_, and 1 for ϵ (in a typical Bayesian regression model), the hierarchical model will sample 44 × 4 priors for *β*′, 4 for *β*_0_, and 1 for ϵ, corresponding to each level in the dataset. Hence, modify the regression equation as follows:

(6) $$y=\beta_{0}\left[level\right]+\beta^{\prime }\left[level\right]\cdot X^{\prime }+\epsilon$$

where level is a hierarchical variable guiding the sampling of the Bayesian model. This variable is not used in the training or testing of the model. It is used to tell the Bayesian sampler to change the sampling space as the level is changed.

(7) $$y=\beta_{0}\left[level\right]+\beta^{\prime }\left[level\right]\cdot X^{\prime }+\epsilon \to for\;level\;0,1,2,3$$

The Bayesian model would then sample different priors with different distribution parameters to learn the posterior for each level. The sampler would run until the model could estimate the best distribution for each level of the LoS, hence capturing the heterogeneity of the target variable. The model specification for HBM is almost similar to that for SBM, with an added sampling layer for each level. Since the estimation equation has an index, ‘*level*’, for sampling different distributions for each hierarchy, the likelihood form for HBM is adopted to incorporate this effect:

(8) $$P\left(y|X^{\prime },level;\beta^{\prime },\beta_{0}\right)=\prod_{level=0}^{3}\prod_{i=1}^{n}{\frac{1}{\sqrt{2\pi }\sigma }e}^{-\frac{1}{2}{\left(\frac{y_{i}-\beta^{\prime }\left[level\right]\cdot {X^{\prime }}_{i}+\beta_{0}\left[level\right])}{\sigma }\right)}^{2}}$$

The coefficients and errors are sampled from normal distributions.

(9) $$\beta^{\prime }\sim Normal\left(\mu_{\beta^{\prime }},\sigma_{\beta^{\prime }}\right),n=44\times number\;of\;levels\left(4\right)\;\beta_{0}\sim Normal\left(\mu_{\beta_{0}},\sigma_{\beta_{0}}\right),n=number\;of\;levels\left(4\right)\;\epsilon \sim Normal\left(\mu_{\epsilon },\sigma_{\epsilon }\right)$$

where $\mu_{\beta^{\prime }}$, $\mu_{\beta_{0}}$, $\sigma_{\beta^{\prime }}$, and $\sigma_{\beta_{0}}$ are the hyperpriors and are sampled from normal and half-normal distributions, respectively.

The learning process is similar to that of SBM. The PyMC package is used to create and train the model with NUTS as a sampler algorithm.

#### 3.3.3. ML Models

The following Table 3 shows the ML models used in this study for LoS prediction.

The implementation of the ML models used in the study is performed using the sklearn library [63] of the Python programming language.

The code used in the study for the creation and testing of the models is available at Github [64].

## 4. Results

The hierarchical Bayesian inference with MCMC on features selected by permutation feature selection resulted in the lowest root mean square error (RMSE) and mean absolute error (MAE) of 1.49 and 1.16, respectively, which is a considerable improvement over the values achieved by ML methods. Table 4 shows the results of the models on testing data benchmarked against various evaluation metrics.

The table shows that all models except SVR were able to estimate the mean of the testing sample with acceptable precision. However, significant differences arise when other statistical measures are analyzed. Only HBM managed to capture the spread of the actual testing data (as seen from the standard deviation values). All the other models failed to capture the variability of the data accurately. The prevalence of extreme values in the data made it difficult for ML models to accurately predict large or exceedingly small values. By analyzing the standard deviations of the predicted values, it is evident that almost no variability in the actual data was estimated by the ML models. ML models under-predicted the standard deviation of the testing sample, which means they could only identify the most frequent values and could not estimate seldom occurring extreme values. A similar phenomenon can be seen by observing each model’s minimum and maximum predicted values (rounded to the nearest whole number).

The coefficient of variance (CV) is a statistical measure of the dispersion of values around the mean. The LoS variable has a CV of 0.43, meaning that most of the values are packed around the mean, with some extreme values in between. If the variable LoS had been completely homogenous, then the coefficient of variance would have been less than 0.20 [65]. The CV for all models is relatively small (smaller than 0.21), which shows that almost all the predicted values (from all ML models along with simple Bayesian regression as well) are present around the mean of the sample while ignoring the presence of extreme values.

Furthermore, the adjusted R-squared for HBM is drastically better than the other models. The exceptionally low adjusted R-squared value for all ML models translates into their inability to capture the variance of the target variable. Lasso shows a negative adjusted R-squared, which means the model could not predict the mean value for most of the testing samples, showing a poor fit.

One interesting trend can be observed by analyzing the RMSE values for the models. All the ML models (except LR) had comparable RMSE (3.2–3.3). Even with hyperparameter tuning, the RMSE did not change, which shows that all these models were ‘stuck’ at the local minima. No performance gains were observed beyond the RMSE of 3.23. Given that most of the values of the target variable lay in the range of 5–8, all the models ended up generating biased predictions around this range. Even Bayesian inference did not show any improvement in the RMSE until the model was changed into a hierarchical regression model (RMSE—1.49).

**Figure 6:**
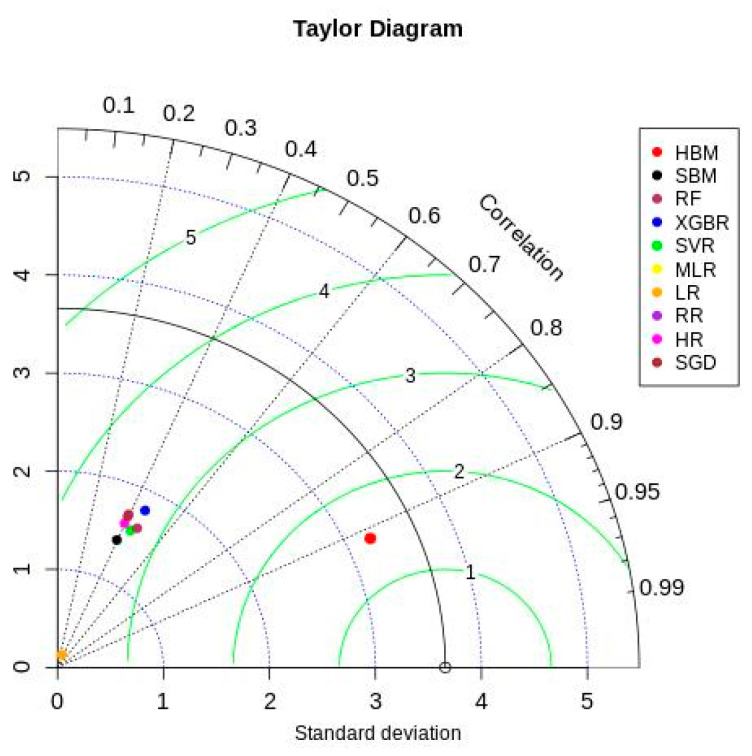
Taylor diagram.

Figure 6 shows the Taylor diagram [66] comparing ML and Bayesian model performances with the testing data. The HBM model showed better results in all three statistics (correlation—0.913, given by the azimuth angle; standard deviation—3.2, given by blue dotted contours; and centered root mean square—1.49, given by green contours). All other models showed relatively lower and similar performance in simulating the observed testing data. These models ended up capturing only the mode of the actual data distribution while underperforming on extreme data points. This adversely affected the ML and SBM model estimates, resulting in their lower performance across all evaluation metrics.

**Figure 7:**
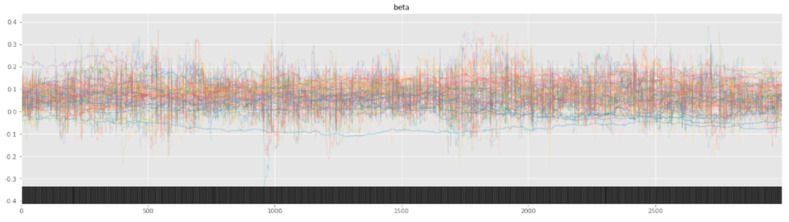
SBM trace plot showing the sampling of (Markov) chains using a NUTS sampler.

**Figure 8:**
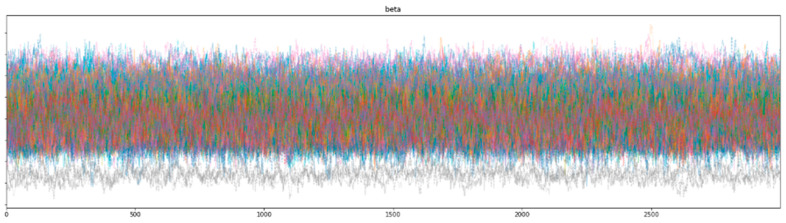
HBM trace plot showing the sampling of (Markov) chains using a NUTS sampler.

Bayesian inference uses a sampler to sample values from specified priors and likelihood to estimate the posterior distribution of the model. The sampling process is crucial for Bayesian models to approximate the parameters. To check the adequacy of the sampling process, a trace plot of the posteriors of the variables is used. Figure 7 and Figure 8 show trace plots of SBM and HBM, respectively.

The sampling process (using a NUTS sampler) uses 4 chains, each with 3000 samples and 1000 tuning steps. Each colored line in Figure 7 and Figure 8 represents the sampling *path* of a chain. At each step, the NUTS sampler chooses a value for each β parameter from their respective priors and accepts or rejects the sampled values based on acceptance probabilities (implemented natively by the NUTS sampler). Consequent sampling of values at each step, along with their acceptance or rejection, creates a sampling path. Figure 8 shows the random *snake-like* movement of the chains, which underlines effective exploration of the parameter space defined by the model priors and hyper-priors. It is important that the chains wander around the parameter space in an ergodic manner to ensure convergence. The significant overlap and mixing of the chains in Figure 8 (*hairy caterpillar* structure) [67] reflects good convergence, which translates into a proper model configuration. Furthermore, the absence of strong trends or jumps in the trace plot of HBM suggests that the MCMC algorithm is efficiently traversing the parameter space, ensuring a more thorough and unbiased exploration.

However, as seen from the trace plot of SBM, the (Markov) chains show irregular sampling with persistent divergences, signifying that the posterior of the parameters varies significantly. In other words, the sampling process did not manage to converge on the correct posteriors for the model parameters, resulting in varying estimations across the chain. The sampling chains in the SBM plot show considerably less mixing and overlap (as compared to the HBM trace plot), along with a few chains showing large jumps with no exploration of the parameter space. This can be attributed to the high variability and skewness of the data. The divergence of the sampling chains warrants model improvements and usually vanishes when a more comprehensive model is specified. This can be seen from the trace plot of HBM having no divergences, signifying the correct model specification.

Another convergence diagnostic is the Gelman Rubin ($\hat{R}$) score [68] (for each parameter), which assesses the variation in the sampled values at the start of the sampling process against the sampled values at the end. The closer the values are to 1, the better the model’s convergence. The $\hat{R}$ score for parameters estimated using SBM showed deviations from the optimum value, signifying that the sampling process did not converge to a stationary distribution, unlike the $\hat{R}$ score for parameters estimated using HBM (given in Appendix B).

## 5. Discussion

The results of this study’s experiments showed that the HBM model outperformed its simpler variant along with other ML models for LoS prediction of cardiac patients undergoing CABG surgery based on their pre-operative variables. Hence, the subsequent part of the discussion explains the estimated coefficients of HBM only (parameter coefficients for other models are given in Appendix C (Table A8, Table A9, Table A10, Table A11, Table A12, Table A13 and Table A14)).

Bayesian estimates of the parameters identified the critical biomarkers affecting the LoS. According to the magnitude and the sign associated with each coefficient value, varying degrees of effects on the target variable can be identified. The HBM estimated four sets of coefficient posteriors for each level of the target variable. Bayesian models output learned distributions of the parameters of the model instead of point estimates. This is useful when greater interpretability of the model is required in terms of causality and uncertainty. The Bayesian models quantify the model’s uncertainty in terms of the deviation/spread of the values in the posterior distributions of the parameters. Table 5a–d shows interval mean values of the estimated parameters.

The four different sets of coefficient values corresponding to different ranges of the LoS variable are useful to identify the varying effects a similar predictor might have on the LoS of a patient. The results of the HBM show that when the LoS of a patient changes from one level to another, the factors contributing to their LoS also change. These changes can be tracked in terms of parameter importance, quantified as the mean of the posterior. These parameter changes conditioned on LoS levels can enable a clinician to assess which factors play a crucial role in determining the lower or higher LoS of a patient, hence offering better explainability than a typical regression model.

Table 5a shows high linearity between a patient’s age and level 0 LoS (between 0–10 days), as cardiac complications are more prevalent in older patients. As age increases, the LoS of a patient also increases. In addition to patient age, arrhythmia, usage of nitrates IV, and pre-operative creatinine levels are the most impactful predictors for level 1 LoS (between 0–10 days) [69]. By analyzing Table 5b of level 1 LoS (between 10–20 days), it is evident that age is still a prominent factor in increasing LoS in cardiac patients. However, the effect of age is not profound, which tells a clinician that there are other factors apart from age that might cause the LoS to increase. *MI_timing* (time between myocardial infraction and CABG procedure), use of ace inhibitors, family history of cardiovascular disease, diastolic BP, pulmonary artery hypertension, *NYHA_class*, and *PCI_timing* (time between incidence of angioplasty and current time) are more prominent factors explaining the LoS between 10–20 days. A total of 44% of patients with LoS between 10–20 days underwent CABG procedures within 1–7 days of the incidence of myocardial infraction, signifying a greater need for patient stabilization before invasive procedures, as compared to only 24% of patients with LoS level 0 (between 0–10) needing additional prep and stabilization times. Similarly, 47% of the patients with LoS level 1 had a higher NYHA risk category as compared to only 29.5% of patients with LoS level 0. In other words, the effect of critical features is more profound at higher LoS levels as compared to lower levels [19].

LoS levels 1 and 2 share quite a few parameters; however, they differ in their magnitudes. The difference in magnitudes suggests that the variable itself takes on larger values and results in a higher contribution towards increasing LoS beyond 20 days. Arrhythmia is a prominent factor in determining the LoS between 0 and 20 days (level 0 and level 1), as seen in Table 5a,b. However, as LoS increases beyond level 1, there are other ‘derived’ factors that become more important; irregular diastolic pressure due to arrhythmia becomes more prevalent in patients with LoS between 20 and 30 days, or abnormal breathing signifies a higher New York Heart Association class of heart failure resulting in level 3 LoS. Additionally, the days required to stabilize and prep the patient before CABG are significantly increased due to the high-risk nature of the patient, coupled with any cardiac complications at the time of admission, resulting in a prolonged LoS (level 3) [70].

Parameter estimation with HBM showed an interesting insight involving the inverse relation between high-risk variables and the LoS. Levels 0 and 1 LoS tend to decrease in the presence of cardiogenic shock. The relationship seems counterintuitive given the high-risk nature of the parameter. However, a deeper analysis of the population showed that patients with a history of cardiogenic shock (or experiencing one just after admission) tend to pass away due to complications, resulting in a shorter stay. Hence, it is clinically relevant. These inverse relationships of high-risk variables with the LoS impart significant information about the mortality risk associated with an inpatient. With these relationships, a new high-risk stratification method can be developed. Clinically relevant variables with a high linear or inverse relationship with LoS can be used to classify the morbidity of cardiac patients, where high-risk patients could be subjected to more extreme medical protocols at an early stage, which can help increase survival chances.

The study compares and evaluates the predictive efficacy of various frequentist and Bayesian regression models to aid and encourage healthcare professionals and researchers to explore the Bayesian paradigm of modeling, especially for LoS, which is highly heterogonous and positively skewed. Most of the literature on LoS prediction uses machine learning models and caters to the variability in the target variable by either removing the outliers or capping the values at a maximum [17]. Another method employed by the researchers is to convert the continuous variable LoS to a dichotomous (early/prolonged) or a categorical variable with multiple bins and ranges (Table 6). This methodology transforms the nature of the problem from a regression to a classification. However, this study treats the LoS as a continuous variable without removing extreme tail values to homogenize the data. The hierarchical Bayesian model of this study outperforms other models from the literature that use ML regression models, with the exception of ref. [15], which reports a low MSE of 0.68 with artificial neural networks. However, the range of the target variable used in the study is short and homogenous: 1–8 days. Whereas, the LoS used in this study ranges from 1 to 65 with high variability.

This study uses only pre-operative clinical features to model the LoS of cardiac patients undergoing CABG. Early identification of LoS is more valuable for healthcare institutions than later predictions. Multiple studies explore LoS prediction based on early indicators with varying degrees of accuracy. However, this study shows the best performance among all others when HBM is used for predictions on the skewed distribution of the target variable.

Very few studies in the literature model the LoS in the Bayesian paradigm. Most notable among these are the studies by Suez-Castillo et al. [29] and Ng et al. [30]. Suez-Castillo et al. propose an asymmetric link function to stratify the incidence of nosomial infection in patients (at North Area Hospital, Jaen, Spain) and its effect on the patient’s length of stay. The researchers compared Bayesian and frequentist estimation of symmetric and asymmetric logistic link function parameters and report an improvement in AUC score with Bayesian estimation of symmetric (AUC—0.96) and asymmetric (AUC—0.99) logit models. The authors extended the study to model the LoS of patients using the Poisson-Gamma model in Bayesian and frequentist paradigms. The authors used non-informative normal priors (similar to this study) for the covariates with the additional novel parameter v to model the randomness (increase or decrease in LoS) resulting from the incidence of nosomial infection after admission. The study used the MCMC method for parameter estimation (similar to this study) and reported sound convergence of the sampling chains. Ng et al. modeled LoS using linear mixture models to accommodate for random effects introduced by the heterogeneity of the target variable and the cohort. The authors used the data of neonates from 23 hospitals across Western Australia from 1998–1999 and modeled the short- and long-term LoS to identify the risk factors. The study used the expectation maximization strategy instead of the MCMC method for the estimation of coefficients and reported impressively lower standard errors. The authors show that prolonged LoS is primarily due to derived factors originating from the sudden complications and severity of the other covariates, which is concordant to the results of this study. Major differences exist in the objective of the study as well as the cohort. This study uses only pre-operative variables for the LoS prediction of cardiac patients; however, both the studies described above used pre- and intra-operative variables as well. Furthermore, the objective of these studies is to model the LoS in terms of its covariates to assess and identify the high-risk factors. Both studies managed to model the data-generating process and report coefficient estimates that closely resembled the original data. However, these studies did not provide out-of-sample predictions of LoS using the learned model coefficients. Furthermore, among all studies, only Suez-Castillo et al. provided a comparative analysis of the results of Bayesian and frequentist models; however, the frequentist methods used are limited and non-trivial (Poisson-Gamma regression model), with no comparison of predictive prowess.

Local studies that predict LoS include Siddiqa et al. [71] and Bajwa et al. [72]. Among these, ref. [72] is a more specialized study carried out in a tertiary care burn center in Lahore, Pakistan. The authors categorized the target variable of LoS into classes and used logistic regression for prediction with an AUC of 0.96. The study uses pre-operative variables for prediction, similar to this study; however, the experiments are limited to ML and other survival models only. Siddiqa et al. used MLR, lasso, ridge, DT, XGB, and RF regression models for LoS prediction on pre- and intra-operative data of patients from the *healthdata.gov* database. The study reports the RF regression model as the best performing one, with an RMSE of 2.23 (MSE—5) as compared to the RMSE of 1.49 achieved in this study using only pre-operative variables modeled using hierarchical Bayesian regression.

## 6. Limitations

The study aims to model the LoS of cardiac patients undergoing CABG surgery while evaluating frequentist and Bayesian regression models to aid and facilitate the healthcare community in exploring the Bayesian paradigm for the LoS problem. The study is carried out on a dataset of one healthcare institution. The study is limited in terms of its cohort. It might be useful to consider the data from multiple health care centers to gauge the changes in predictive accuracy of Bayesian and frequentist methods in the presence of increased data size. Bayesian models generalize better to the target population; however, they have high complexity in terms of their design and execution times. The training time increases with the addition of more data; hence, a tradeoff between runtime and accuracy is usually needed.

Moreover, the study uses only pre-operative variables for early LoS prediction, which is more desirable as earlier estimates of a patient’s stay help institutes with timely resource and personnel allocation. However, this limits the accuracy of the model predictions as more definitive and critical factors are mostly available peri- or post-operatively. This tradeoff between earlier versus accurate predictions can be solved by using a dynamic model, which can update its predictions with the advent of new data. This would lead to better visibility of the patient’s hospital stay through various stages of hospitalization. Hierarchical Bayesian regression offers interpretability in terms of changing coefficient values across different levels/hierarchies. By analyzing the changes in magnitude and direction (sign) of a variable across various hierarchies, useful insights can be obtained.

Future work can be conducted to explore the use of the Shapley Additive Explanations (SHAP) paradigm of interpretation in Bayesian models. SHAP is not directly applicable to Bayesian models (especially hierarchical models) because they produce posterior distributions for the coefficients instead of point estimates. Hence, calculating SHAP values for each sample in the posterior distribution would be computationally expensive. However, research in the area of integrating open source Bayesian modeling libraries (PyMC and Stan) to support SHAP natively is an interesting and profound area of interest, with applications extending to the healthcare domain.

## 7. Conclusions

Hierarchical variants of Bayesian inference with MCMC methods have proven to be useful for predicting LoS in cardiac patients based on their pre-operative variables. The accuracy shown by the hierarchical Bayesian regression model is better (RMSE—1.49, MAE—1.16) than that of ML models when data variability is high with extreme values, with the added advantage of greater interpretability. From the results of this study, it is evident that a simple Bayesian regression model without hierarchy is insufficient to explain the variability of the data.

Furthermore, many ML models also fell short of capturing the real variance of the dataset, especially when the data are highly volatile. The HBM is especially useful when the target variable has different value ranges. ‘Soft’ division of the dataset into groups or levels (by indexing) can be used to create a hierarchy of heterogeneous, continuous variables. A separate set of parameter values for each level in the LoS variable helps assess the effect of clinical variables as LoS increases in a patient. Analyzing the varying effects of the same variable across different LoS ranges will help the clinician plan the appropriate intervention.

By using Bayesian inference quantification (with hierarchical changes) of the parameter, medical staff can create a mechanism of risk stratification where the high-risk features affecting the LoS the most (linearly or inversely) can be known beforehand. This will enable clinicians and administrative staff to look for these factors in an in-patient, classify them as high-risk, and release resources accordingly. Bayesian inference methods (especially hierarchical Bayesian models) are computationally extensive but result in good estimates with better clinical explainability.

## Figures and Tables

**Table 1:** (a) Dataset (continuous variables). (b) Dataset (categorical variables).

**(a) Dataset (Continuous Variables)**						
**Feature**	**Description**	**Type**	**Min**	**Max**	**Average**	**Std**
*admission_to_surgery*	Admission to surgery (Days)	Continuous	0	21	2.26	1.94
*LOS_Surgery_to_discharge*	Surgery to discharge (Days)	Continuous	0	59	6.04	2.97
*last_wbc_count*	Last WBC count (×10^9^/L)	Continuous	2.8	48.8	9.48	2.77
*BMI*	BMI (kg/m^2^)	Continuous	14.82	47.08	26.82	4.35
*Patient_age*	Age of patient (Years)	Continuous	23	90	58.07	8.66
*last_hematocrit*	Last hematocrit value (%)	Continuous	21.2	56.2	40.51	4.92
*last_cretenine_preop*	Last creatinine value (mg/dL)	Continuous	0.41	13.3	1.14	0.63
*BPsystolic*	Pre-operative systolic BP (mmHg)	Continuous	76	199	122.49	15.94
*diastolic*	Pre-operative diastolic BP (mmHg)	Continuous	27	117	70.36	9.63
*ejection_fraction*	Pre-operative LV ejection fraction (%)	Continuous	10	65	42.89	11.18
*weight*	Weight of the patient (kg)	Continuous	36	128	71.87	12.74
height	Height of the patient (cm)	Continuous	123	191	163.6	8.33
**(b) Dataset (Categorical Variables)**						
**Feature**	**Description**	**Type**	**Labels**		**n (%)**	
*gender_id*	Gender of patient	Categorical	Male, Female		4360 (82), 954 (17.9)	
*pulmonary_artery_done*	Test for pulmonary artery mean pressure conducted?	Categorical	Yes, No		2205 (41.4), 3109 (58.5)	
*Active_tobacco_use*	Tobacco use within the last 6 months	Categorical	Yes, No		1308 (25.8), 3975 (74.1)	
*f_history_cad*	Family history of coronary artery disease	Categorical	Yes, No		2664 (49.6), 2699 (50.3)	
*diabetes*	Diabetes/insulin use	Categorical	No, Yes (Non-Insulin Dependent, Yes (Insulin Dependent)		2067 (38.5), 2211 (41.2), 1085 (20.3)	
*myocardial_infarction*	Any prior myocardial infarction (MI)	Categorical	Yes, No		3816 (71.1), 1547 (28.8)	
*MI_timing*	Time between MI and CABG	Categorical	No MI, <6 h, >6–24 h, 1–7 days, 8–21 days, >21 days		1584 (29.5), 16 (0.29), 26 (0.48), 1473 (27.4), 938 (17.4), 1326 (24.7)	
*congestive_heart_failure_A*	Congestive heart failure	Categorical	Yes, No		471 (8.7), 4892 (91.2)	
*NYHA_class*	NYHA (New York Heart Association) shortness of breath class during the last 2 weeks	Categorical	Not applicable, NYHA I, NYHA II, NYHA III, NYHA IV		2482 (46.2), 62 (1.1), 1059 (19.7), 1513 (28.2), 247 (4.6)	
*Cardiac_Presentation_on_Admission*	Cardiac symptoms on arrival	Categorical	No Symptoms of Angina, Symptoms but unlikely to be ischemic, Stable Angina, Unstable Angina, Non-ST Elevation MI, ST Elevation MI		301 (5.6), 370 (6.8), 575 (10.7), 1664 (31.0), 1778 (33.1), 675 (12.5)	

**Table 2:** List of features from the permutation feature importance method.

*patient_age*	*arrhythmia*	*Arrhythmia Type Sust VT/VF*
*last_wbc_count*	*lipid_lowering*	*myocardial_infarction*
*last_cretenine_preop*	*Prior_PCI*	*resuscitation*
*BPsystolic*	*Cerebovascular_disease*	*dialysis*
*last_hematocrit*	*Pulmonary_insuff*	*steroids*
*Cardiac_Presentation_on_Admission*	*MI_timing*	*previous_coronary_bypass*
*Statin*	*inotropes*	*previous_valve*
*Mitral_regurgitation*	*Angina_class*	*FirstCVSurgery*
*diastolic*	*PCI_timing*	*warfarin*
*gender_id*	*congestive_heart_failure*	*ace_inhibitors*
*CABG_status*	*adp_inhibitors_within_5days*	*Carotid_disease*
*nitratesIV*	*cardiogenic_shock*	*aspirin*
*family_history_of Cardiac_disease*	*intracardiac_device*	*left_main_disease*
*beta_blockers*	*pulmonary_artery_hypertension*	*bronchodilators*
	*NYHA_class*	*Coronaries_diseased*

**Table 3:** ML models.

Model	Hyperparameters
Stochastic Gradient Descent Regression	learning rate: adaptive, inverse scaling factor: 0.899, regularization parameter: 0.890
Huber Regression [57]	k: 4
XGBoost Regression [58]	subsample: 0.8, number of estimators: 1800, minimum sample split: 5, minimum samples leaf: 4, minimum child weight: 6, maximum features: auto, maximum depth: 68, learning rate: 0.01, column sample by tree: 0.2, booster: gbtree, alpha: 0.8, lambda: 0.8
Random Forest Regression [59]	number of estimators: 1200, minimum sample split: 10, minimum sample leaf: 4, maximum features: sqrt, maximum depth: 20, bootstrap: False.
Lasso Regression [60]	λ: 0.01
Ridge Regression [61]	λ: 1.08
Support Vector Regression [62]	kernel: polynomial, degree: 2, regularization: 0.3
Multiple Linear Regression	

**Table 4:** Statistical summary of the target variable—actual and estimated.

	Mean	Standard Deviation	Min	Max	CV	Adjusted R-Squared	RMSE	MAE
Actual	8.37	3.65	1	47	0.43	-	-	-
HBM	8.32	3.23	4	40	0.38	82.3	1.49	1.16
SBM	8.31	1.41	5	16	0.17	11.9	3.36	2.05
XGB	8.36	1.80	6	16	0.21	17.4	3.25	1.88
RF	8.34	1.60	6	15	0.19	18.4	3.23	1.87
SVR	7.66	1.55	5	15	0.20	11.9	3.36	1.85
Lasso	8.28	0.13	8	9	0.01	−2.15	3.61	2.28
Ridge	8.36	1.69	5	16	0.20	11.4	3.37	2.00
SGD	8.35	1.67	5	15	0.20	11.4	3.36	2.00
HR	8.26	1.59	5	15	0.19	11.6	3.36	1.98
MLR	8.36	1.70	5	16	0.20	11.3	3.37	2.00

**Table 5:** (a) Estimated parameter coefficient value (level 0). (b) Estimated parameter coefficient value (level 1). (c) Estimated parameter coefficient value (level 2). (d) Estimated parameter coefficient value (level 3).

**(a) Estimated Parameter Coefficient Value (Level 0)**					
**Parameter**	**θ ± sd**	**Parameter**	**θ ± sd**	**Parameter**	**θ ± sd**
$\beta_{\;cardiogenic\_shock}$	−0.348 ± 0.20	$\beta_{\;previous\_coronary\_bypass}$	0.074 ± 0.19	$\beta_{\;intracardiac\_device}$	−0.035 ± 0.18
$\beta_{\;age}$	0.242 ± 0.08	$\beta_{\;FirstCVSurgery}$	0.073 ± 0.19	$\beta_{\;dialysis}$	0.03 ± 0.18
$\beta_{\;arrhythmia}$	0.212 ± 0.16	$\beta_{\;Carotid\_disease}$	0.073 ± 0.18	$\beta_{\;pulmonary\_insuff}$	−0.03 ± 0.06
$\beta_{\;nitratesIV}$	0.196 ± 0.1	$\beta_{\;aspirin}$	0.067 ± 0.09	$\beta_{\;Arrhythmia\;Type\;Sust\;VT\;/\;VF}$	−0.029 ± 0.18
$\beta_{\;last\_cretenine\_preop}$	0.178 ± 0.06	$\beta_{\;NYHA\_class}$	0.064 ± 0.02	$\beta_{\;ace\_inhibitors\_}$	−0.017 ± 0.07
$\beta_{\;left\_main\_disease}$	0.16 ± 0.07	$\beta_{\;previous\_valve}$	0.06 ± 0.19	$\beta_{\;last\_hematocrit}$	−0.016 ± 0.03
$\beta_{\;myocardial\_infarction}$	0.144 ± 0.14	$\beta_{\;pulmonary\_artery\_hypertension}$	0.059 ± 0.17	$\beta_{\;MI\_timing}$	−0.014 ± 0.03
$\beta_{\;Cerebovascular\_disease}$	0.139 ± 0.12	$\beta_{\;Angina\_class}$	0.058 ± 0.02	$\beta_{\;PCI\_timing}$	−0.002 ± 0.09
$\beta_{\;beta\_blockers}$	−0.136 ± 0.09	$\beta_{\;Coronaries\_diseased}$	−0.055 ± 0.09	$\beta_{\;congestive\_heart\_failure}$	0.001 ± 0.18
$\beta_{\;adp\_inhibitors\_within\_5days}$	0.129 ± 0.10	$\beta_{\;steroids}$	0.052 ± 0.17	$\beta_{\;last\_wbc\_count}$	−0.001 ± 0.03
$\beta_{\;resuscitation}$	−0.126 ± 0.19	$\beta_{\;gender}$	0.044 ± 0.03		
$\beta_{\;inotropes}$	−0.113 ± 0.16	$\beta_{\;Cardiac\_Presentation\_on\_Admission}$	−0.043 ± 0.03		
$\beta_{\;lipid\_lowering}$	0.109 ± 0.13	$\beta_{\;Statin}$	0.042 ± 0.13		
$\beta_{\;family\_history\_of\_coronary\_artery\_disease}$	−0.108 ± 0.06	$\beta_{\;diastolic}$	0.041 ± 0.04		
$\beta_{\;CABG\_status}$	0.089 ± 0.06	$\beta_{\;bronchodilators}$	0.04 ± 0.14		
**(b) Estimated Parameter Coefficient Value (Level 1)**					
**Parameter**	**θ ± sd**	**Parameter**	**θ ± sd**	**Parameter**	**θ ± sd**
$\beta_{\;ace\_inhibitors}$	0.388 ± 0.18	$\beta_{\;CABG\_status}$	−0.12 ± 0.16	$\beta_{\;intracardiac\_device}$	0.06 ± 0.18
$\beta_{\;family\_history\_of\_coronary\_artery\_disease}$	0.326 ± 0.18	$\beta_{\;last\_wbc\_count}$	−0.11 ± 0.13	$\beta_{\;steroids}$	0.06 ± 0.18
$\beta_{\;PCI\_timing}$	0.317 ± 0.17	$\beta_{\;aspirin}$	0.11 ± 0.17	$\beta_{\;previous\_coronary\_bypass}$	0.06 ± 0.18
$\beta_{\;diastolic}$	0.305 ± 0.16	$\beta_{\;myocardial\_infarction}$	0.10 ± 0.18	$\beta_{\;warfarin}$	0.06 ± 0.19
$\beta_{\;arrhythmia}$	0.277 ± 0.19	$\beta_{\;left\_main\_disease}$	0.09 ± 0.17	$\beta_{\;Prior\_PCI}$	−0.05 ± 0.18
$\beta_{\;MI\_timing}$	0.277 ± 0.09	$\beta_{\;patient\_age}$	0.08 ± 0.13	$\beta_{\;adp\_inhibitors\_within\_5days}$	−0.05 ± 0.17
$\beta_{\;pulmonary\_artery\_hypertension}$	0.241 ± 0.2	$\beta_{\;Statin}$	−0.08 ± 0.18	$\beta_{\;last\_hematocrit}$	0.03 ± 0.12
$\beta_{\;NYHA\_class}$	0.235 ± 0.1	$\beta_{\;Mitral\_regurgitation}$	−0.08 ± 0.14	$\beta_{\;Coronaries\_diseased}$	0.01 ± 0.18
$\beta_{\;Cerebovascular\_disease}$	0.21 ± 0.19	$\beta_{\;FirstCVSurgery}$	0.08 ± 0.18	$\beta_{\;pulmonary\_insuff}$	0.01 ± 0.16
$\beta_{\;Cardiac\_Presentation\_on\_Admission}$	−0.19 ± 0.12	$\beta_{\;previous\_valve}$	0.07 ± 0.19	$\beta_{\;last\_cretenine\_preop}$	−0.01 ± 0.17
$\beta_{\;beta\_blockers}$	0.175 ± 0.17	$\beta_{\;inotropes}$	0.07 ± 0.19		
$\beta_{\;gender}$	0.147 ± 0.17	$\beta_{\;Carotid\_disease}$	0.07 ± 0.19		
$\beta_{\;nitratesIV}$	0.145 ± 0.18	$\beta_{\;bronchodilators}$	0.07 ± 0.19		
$\beta_{Arrhythmia\;Type\;Sust\;VT\;/\;VF}$	0.137 ± 0.18	$\beta_{\;lipid\_lowering}$	−0.07 ± 0.18		
$\beta_{\;Angina\_class}$	0.134 ± 0.09	$\beta_{\;resuscitation}$	0.07 ± 0.19		
$\beta_{\;congestive\_heart\_failure}$	0.133 ± 0.17	$\beta_{\;cardiogenic\_shock}$	0.072 ± 0.19		
$\beta_{\;dialysis}$	0.072 ± 0.19	$\beta_{\;BPsystolic}$	−0.006 ± 0.12		
**(c) Estimated Parameter Coefficient Value (Level 2)**					
**Parameter**	**θ ± sd**	**Parameter**	**θ ± sd**	**Parameter**	**θ ± sd**
$\beta_{\;MI\_timing}$	0.199 ± 0.17	${\beta \;}_{gender}$	0.077 ± 0.19	$\beta_{\;Cardiac\_Presentation\_on\_Admission}$	0.060 ± 0.19
$\beta_{\;diastolic}$	0.158 ± 0.18	$\beta_{\;Cerebovascular\_disease}$	0.077 ± 0.18	$\beta_{\;Angina\_class}$	0.062 ± 0.18
${\beta \;}_{\;ace\_inhibitors}$	0.109 ± 0.19	$\beta_{\;Mitral\_regurgitation}$	0.075 ± 0.18	$\beta_{\;inotropes}$	0.061 ± 0.19
$\beta_{\;beta\_blockers}$	0.105 ± 0.18	$\beta_{\;resuscitation}$	0.075 ± 0.19	$\beta_{\;cardiogenic\_shock}$	0.059 ± 0.19
$\beta_{\;lipid\_lowering}$	0.104 ± 0.19	$\beta_{\;First\_cardiovasular\_Surgery}$	0.075 ± 0.19	$\beta_{\;Prior\_PCI}$	0.058 ± 0.19
$\beta_{\;Statin}$	0.1 ± 0.18	$\beta_{\;pulmonary\_insuff}$	0.074 ± 0.19	$\beta_{\;nitratesIV}$	0.053 ± 0.19
$\beta_{\;aspirin}$	0.097 ± 0.19	$\beta_{\;warfarin}$	0.074 ± 0.19	$\beta_{\;congestive\_heart\_failure}$	0.049 ± 0.18
$\beta_{\;last\_cretenine\_preop}$	0.092 ± 0.19	$\beta_{\;intracardiac\_device}$	0.072 ± 0.19	$\beta_{\;family\_history\_of\_coronary\_artery\_disease}$	0.048 ± 0.19
$\beta_{\;left\_main\_disease}$	0.092 ± 0.18	$\beta_{\;Arrhythmia\;Type\;Sust\;VT\;/\;VF}$	0.072 ± 0.18	$\beta_{\;arrhythmia}$	0.048 ± 0.19
$\beta_{\;Coronaries\_diseased}$	0.09 ± 0.19	$\beta_{\;previous\_valve}$	0.071 ± 0.19	$\beta_{\;CABG\_status}$	0.021 ± 0.19
$\beta_{\;myocardial\_infarction}$	0.089 ± 0.18	$\beta_{\;Carotid\_disease}$	0.071 ± 0.19		
$\beta_{\;BPsystolic}$	0.086 ± 0.18	$\beta_{\;bronchodilators}$	0.071 ± 0.19		
$\beta_{\;PCI\_timing}$	0.084 ± 0.18	$\beta_{\;adp\_inhibitors\_within\_5days}$	0.069 ± 0.18		
$\beta_{\;patient\_age}$	0.082 ± 0.19	$\beta_{\;pulmonary\_artery\_hypertension}$	0.069 ± 0.18		
$\beta_{\;NYHA\_class}$	0.082 ± 0.19	$\beta_{\;previous\_coronary\_bypass}$	0.069 ± 0.19		
**(d) Estimated Parameter Coefficient Value (Level 3)**					
**Parameter**	**θ ± sd**	**Parameter**	**θ ± sd**	**Parameter**	**θ ± sd**
$\beta_{\;Cardiac\_Presentation\_on\_Admission}$	0.103 ± 0.19	$\beta_{\;left\_main\_disease}$	0.075 ± 0.19	$\beta_{\;inotropes}$	0.068 ± 0.19
$\beta_{\;NYHA\_class}$	0.099 ± 0.19	$\beta_{\;congestive\_heart\_failure}$	0.074 ± 0.19	$\beta_{\;family\_history\_of\_coronary\_artery\_disease}$	0.067 ± 0.19
${\beta \;}_{\;Angina\_class}$	0.097 ± 0.19	$\beta_{\;myocardial\_infarction}$	0.074 ± 0.19	$\beta_{\;First\_cardiovascular\_Surgery}$	0.067 ± 0.19
$\beta_{\;nitratesIV}$	0.087 ± 0.18	$\beta_{\;previous\_coronary\_bypass}$	0.074 ± 0.18	$\beta_{\;ace\_inhibitors}$	0.066 ± 0.18
$\beta_{\;MI\_timing}$	0.087 ± 0.19	$\beta_{\;warfarin}$	0.074 ± 0.19	$\beta_{\;pulmonary\_artery\_hypertension}$	0.064 ± 0.19
$\beta_{\;Statin}$	0.084 ± 0.19	$\beta_{\;patient\_age}$	0.073 ± 0.18	$\beta_{\;resuscitation}$	0.063 ± 0.19
$\beta_{\;last\_cretenine\_preop}$	0.083 ± 0.19	$\beta_{\;cardiogenic\_shock}$	0.073 ± 0.19	$\beta_{\;steroids}$	0.063 ± 0.19
$\beta_{\;lipid\_lowering}$	0.083 ± 0.19	$\beta_{\;previous\_valve}$	0.073 ± 0.18	$\beta_{\;BPsystolic}$	0.061 ± 0.19
$\beta_{\;PCI\_timing}$	0.083 ± 0.18	$\beta_{\;last\_wbc\_count}$	0.072 ± 0.18	$\beta_{\;diastolic}$	0.059 ± 0.19
$\beta_{\;arrhythmia}$	0.081 ± 0.18	$\beta_{\;CABG\_status}$	0.072 ± 0.19	$\beta_{\;last\_hematocrit}$	0.057 ± 0.19
$\beta_{\;bronchodilators}$	0.081 ± 0.19	$\beta_{\;dialysis}$	0.072 ± 0.18		
$\beta_{\;aspirin}$	0.08 ± 0.19	$\beta_{\;Prior\_PCI}$	0.071 ± 0.19		
$\beta_{\;Coronaries\_diseased}$	0.078 ± 0.19	$\beta_{\;adp\_inhibitors\_within\_5days}$	0.07 ± 0.18		
$\beta_{\;gender}$	0.077 ± 0.2	$\beta_{\;intracardiac\_device}$	0.07 ± 0.19		
$\beta_{\;beta\_blockers}$	0.077 ± 0.18	$\beta_{Arrhythmia\;Type\;Sust\;VT\;/\;VF}$	0.069 ± 0.19		

**Table 6:** Comparing studies from the literature where the objective is LoS prediction and at least a single learning model and its findings are reported.

Study	Models	Target Type	Variables	Metric	Results
[1]	ANN, Classification Trees, Tree Bagger, RF, Fuzzy Logic, SVM, KNN, Regression Trees, Naïve Bayes	Classification	Pre-op	Accuracy	63.21%, 62.90%, 59.89%, 60.21%, 57.56%, 61.89%, 56.95%, 65.86%
[12]	RF, ANN	Classification	Pre-op	Accuracy	92%, 95%
[15]	MR, LR, SGD, Elastic Net, Linear SVM, KNN, DT, RF, AdaBoost, XGB, Scikit MLP, PyTorch MLP	Regression	Pre-op	MSE	0.9, 0.78, 0.78, 0.78, 0.77, 0.96, 0.88, 0.82, 0.84, 0.82, 0.78, 0.68
[16]	LR, Gradeint Boosting Regression, RF, SG	Regression	Pre-op	RMSE	2.43, 1.97, 1.96, 2.46
[17]	ANN, SVM, PCR	Regression	Intra-op	MAE	3.0, 2.5, 2.14
[18]	DT, ANN, SVM, Ensemble	Classification	Pre-op	Accuracy	83.5%, 53.9%, 96.4%, 95.9%
[20]	ANN	Classification	Pre-op	AUC	0.9
[21]	ANN	Classification	Intra-op	ROC	0.69
[22]	RF, SVM, SVM (Learning Using Previliged Information), MTL (Multi-Task Learning), MLR	Classification	Intra-op	AUC	0.70, 0.74, 0.76, 0.56, 0.45
[23]	DT, SVM, RF	Classification	Intra-op	Accuracy	0.75, 0.81, 0.87
[24]	LR	Regression	Intra-op	MAPE	17.65, 20.12. 22.45, 22.01, 21.84
[27]	Bayesian Network	Classification	Intra-op	AUC	0.83
[28]	Bayesian Network	Classification	Intra-op	Accuracy	80%
[71]	MLR, Lasso, Ridge, DTR, XGB, RF	Regression	Intra-op	MSE	38.49, 42.19, 38.49, 5.93, 5.62, 5
[72]	Logistic Regression	Classification	Intra-op	AUC	0.82

## Data Availability

Data are contained within the article.

## Appendix A. Dataset Overview

**Table A1:** Variable description (Continuous).

Feature	Description	Type	Min	Max	Average	Std	IQR	% of Missing Values
*TempSNO*	Serial number	Incremental						
*date_of_admission*	Admission date	datetime	25 November 2015	30 December 2020				0
*date_of_surgery*	Surgery date	datetime	28 November 2015	31 December 2020				0
*date_of_discharge*	Discharge date	datetime	5 December 2015	7 January 2021				0
*patient_age*	Age of patient (Years)	Continuous	23	90	58.07	8.66	12	0
*Admission_to_surgery*	Admission to surgery (Days)	Continuous	0	21	2.26	1.94	2	0
*LOS_Surgery_to_discharge*	Surgery to discharge (Days)	Continuous	0	59	6.04	2.97	1	0
*last_wbc_count*	Last WBC count (×10^9^/L)	Continuous	2.8	48.8	9.48	2.77	3.1	0.41
*BMI*	BMI (kg/m^2^)	Continuous	14.82	47.08	26.82	4.35	5.47	0.41
*last_hematocrit*	Last hematocrit value (%)	Continuous	21.2	56.2	40.51	4.92	6.6	0
*last_cretenine_preop*	Last creatinine value (mg/dL)	Continuous	0.41	13.3	1.14	0.63	0.32	0
*BPsystolic*	Pre-operative systolic BP (mmHg)	Continuous	76	199	122.49	15.94	23	0.48
*diastolic*	Pre-operative diastolic BP (mmHg)	Continuous	27	117	70.36	9.63	14	0.41
*ejection_fraction*	Pre-operative LV ejection fraction (%)	Continuous	10	65	42.89	11.18	20	0
*Weight*	Weight of the patient (kg)	Continuous	36	128	71.87	12.74	17	0
*Height*	Height of the patient (cm)	Continuous	123	191	163.6	8.33	10	0

**Table A2:** Variable description (Categorical).

Feature	Description	Type	Labels	n (%)	% of Missing Values
*gender_id*	Gender of patient	Categorical	Male, Female	4360 (82), 954 (17.9)	
*pulmonary_artery_done*	Test for pulmonary artery mean pressure conducted?	Categorical	Yes, No	2205 (41.4), 3109 (58.5)	0.41
*Active_tobacco_use*	Tobacco use within last 6 months	Categorical	Yes, No	1308 (25.8), 3975 (74.1)	
*f_history_cad*	Family history of coronary artery disease	Categorical	Yes, No	2664 (49.6), 2699 (50.3)	
*diabetes*	Diabetes/insulin use	Categorical		2067 (38.5), 2211 (41.2), 1085 (20.3)	
*myocardial_infarction*	Any prior myocardial infarction (MI)	Categorical	Yes, No	3816 (71.1), 1547 (28.8)	
*MI_timing*	Time between MI and CABG	Categorical		1584 (29.5), 16 (0.29), 26 (0.48), 1473 (27.4), 938 (17.4), 1326 (24.7)	
*congestive_heart_failure_A*	Congestive heart failure	Categorical	Yes, No	471 (8.7), 4892 (91.2)	
*NYHA_class*	NYHA (New York Heart Association shortness of breath class) during last 2 weeks	Categorical		2482 (46.2), 62 (1.1), 1059 (19.7), 1513 (28.2), 247 (4.6)	
*Cardiac_Presentation_on_Admission*	Cardiac symptoms on arrival	Categorical		301 (5.6), 370 (6.8), 575 (10.7), 1664 (31.0), 1778 (33.1), 675 (12.5)	
*Angina_class*	Angina Canadian Cardiac Society (CCS) classification within last 2 weeks	Categorical		1069 (19.7), 26 (0.48), 700 (13.0), 2443 (45.5), 1135 (21.1)	
*cardiogenic_shock*	Cardiogenic shock at the time of CABG	Categorical	Yes, No	65 (1.21), 5298 (98.7)	
*resuscitation*	Any cardiac resuscitation within one hour of CABG	Categorical	Yes, No	15 (0.27), 5348 (99.7)	
*arrhythmia*	Any prior arrhythmia	Categorical	Yes, No	122 (2.27), 5241 (97.7)	
*SustVTVF*	Sustained VT/VF within 2 weeks	Categorical	Yes, No	60 (1.11), 5303 (98.8)	
*AFibFlutter*	Afib/Flutter within 2 weeks	Categorical	None, Afib/Flutter, Heart block	5298 (98.7), 51 (0.95), 14 (0.26)	
*ventilator_used*	Patient already on ventilator	Categorical	Yes, No	76 (1.41), 5287 (98.5)	
*beta_blockers_A*	Beta blockers within 24 h	Categorical	Yes, No	4593 (85.6), 770 (14.3)	
*ace_inhibitors_A*	ACE inhibitors within 24 h	Categorical	Yes, No	1116 (20.8), 4247 (79.1)	
*nitratesIV*	Nitrates I.V at time of CABG	Categorical	Yes, No	553 (10.3), 4810 (89.6)	
*Angina_class*	Angina Canadian Cardiac Society (CCS) classification within last 2 weeks	Categorical		1069 (19.7), 26 (0.48), 700 (13.0), 2443 (45.5), 1135 (21.1)	
*cardiogenic_shock*	Cardiogenic shock at the time of CABG	Categorical	Yes, No	65 (1.21), 5298 (98.7)	
*resuscitation*	Any cardiac resuscitation within one hour of CABG	Categorical	Yes, No	15 (0.27), 5348 (99.7)	
*arrhythmia*	Any prior arrhythmia	Categorical	Yes, No	122 (2.27), 5241 (97.7)	
*SustVTVF*	Sustained VT/VF within 2 weeks	Categorical	Yes, No	60 (1.11), 5303 (98.8)	
*AFibFlutter*	Afib/Flutter within 2 weeks	Categorical	None, Afib/Flutter, Heart block	5298 (98.7), 51 (0.95), 14 (0.26)	
*ventilator_used*	Patient already on ventilator	Categorical	Yes, No	76 (1.41), 5287 (98.5)	
*beta_blockers_A*	Beta blockers within 24 h	Categorical	Yes, No	4593 (85.6), 770 (14.3)	
*ace_inhibitors_A*	ACE inhibitors within 24 h	Categorical	Yes, No	1116 (20.8), 4247 (79.1)	
*nitratesIV*	Nitrates I.V at time of CABG	Categorical	Yes, No	553 (10.3), 4810 (89.6)	
*anti_coagulants*	Anticoagulants within 48 h prior to surgery	Categorical	No, UFH, LMWH, Thrombin inhibitors	4061 (75.7), 924 (17.2), 375 (6.9), 3 (0.05)	
*warfarin_A*	Warfarin within 24 h of CABG	Categorical	Yes, No	5254 (97.9), 109 (2.1)	
*inotropes*	Inotropes within 48 h	Categorical	Yes, No	76 (1.4), 5287 (98.6)	
*steroids*	Steroids witihn 24 h	Categorical	Yes, No	5 (0.1), 5358 (99.9)	
*aspirin_A*	Aspirin within 5 days of CABG	Categorical	Yes, No	4758 (88.7), 605 (11.3)	
*lipid_lowering_A*	Any lipid-lowering medications within 24 h of CABG	Categorical	Yes, No	4696 (87.6), 667 (12.4)	
*Dyslipidemia*	Dyslipidemia with statins	Categorical	No, Yes (On Statin), Yes (Not on Statin)	11 (0.2), 3254 (60.7), 2091 (39.0), 7 (0.1)	
*dialysis*	Hemodialysis-dependent pre-operatively	Categorical	Yes, No	42 (0.8), 5321 (99.2)	
*hypertension*	Hypertension	Categorical	Yes, No	3859 (72.0), 1504 (28.0)	
*Cerebovascular_disease*	Any cerebrovascular disease	Categorical	Yes, No	283 (5.3), 5080 (94.7)	
*pulmonary_insuff*	Pulmonic insufficiency	Categorical	None, Trivial, Mild, Moderate, Severe	4705 (87.7), 438 (8.2), 188 (3.5), 6 (0.1), 4 (0.1)	
*Carotid_disease*	Carotid disease	Categorical	Yes, No	7 (0.1), 5356 (99.9)	
*chronic_lung_disease*	Chronic lung disease	Categorical	Yes, No	119 (2.2), 5244 (97.8)	
*FirstCVSurgery*	Incidence of CV surgery	Categorical	First CV Surgery, 2nd CV surgery, 3rd CV surgery, 4th CV surgery	5341 (99.6), 19 (0.4), 2 (0.0), 1 (0.0)	
*previous_cv_interventions*	Previous CV interventions (prior PCI, CABG, or others)	Categorical	Yes, No	720 (13.4), 4643 (86.6)	
*previous_coronary_bypass*	Previous coronary bypass (CABG)	Categorical	Yes, No	21 (0.4), 5342 (99.6)	
*previous_valve*	Previous valve surgery	Categorical	Yes, No	1 (0.0), 5362 (100.0)	
*intracardiac_device*	Previous intracardiac device pacemaker or defibrillator (PPM or ICD) usage	Categorical	Yes, No	11 (0.2), 5352 (99.8)	
*Prior_PCI*	Any previous angioplasty (PCI)	Categorical	Yes, No	696 (13.0), 4667 (87.0)	
*PCI_timing*	PCI interval: within or more than 6 h	Categorical	>6 h, <6 h, No PCI	690 (12.9), 6 (0.1), 4667 (87.0)	
*Statin_A*	Statin within 24 h	Categorical	Yes, No	4695 (87.5), 668 (12.5)	
*adp_inhibitors_within_5days*	ADP inhibitors within five days	Categorical	Yes, No	392 (7.3), 4971 (92.7)	
*bronchodilators*	Routine use of bronchodilators	Categorical	Yes, No	86 (1.6), 5277 (98.4)	
*Coronaries_diseased*	Number of diseased coronary arteries	Categorical	Yes, No	4842 (90.3), 521 (9.7)	
*left_main_disease*	Left main coronary disease	Categorical	Yes, No	1007 (18.8), 4356 (81.2)	
*pulmonary_artery_hypertension*	Severe pulmonary artery hypertenstion (PASP 65 mmHg or more)	Categorical	Yes, No	31 (0.6), 5332 (99.4)	
*Aortic_regurgitation*	Aortic insufficiency	Categorical	None, Mild, Moderate	4958 (92.4), 375 (7.0), 30 (0.6)	
*Mitral_regurgitation*	Mitral insufficiency	Categorical	None, Mild, Moderate, Severe	2571 (47.9), 1960 (36.5), 790 (14.7), 42 (0.8)	
*Tricuspid_regurgitation*	Tricuspid insufficiency	Categorical	None, Mild, Moderate, Severe	3802 (70.9), 1560 (29.1), 1 (0.0)	
*CABG_status*	CABG surgery status	Categorical	Elective, Urgent, Emergent, Emergent Salvage	3049 (56.9), 2082 (38.8), 217 (4.0), 15 (0.3)	
*resuscitation*	Any cardiac resuscitation within one hour of CABG	Categorical	Yes, No	15 (0.27), 5348 (99.7)	
*arrhythmia*	Any prior arrhythmia	Categorical	Yes, No	122 (2.27), 5241 (97.7)	
*SustVTVF*	Sustained VT/VF within 2 weeks	Categorical	Yes, No	60 (1.11), 5303 (98.8)	
*AFibFlutter*	Afib/Flutter within 2 weeks	Categorical	None, Afib/Flutter, Heart block	5298 (98.7), 51 (0.95), 14 (0.26)	
*City*	Patient City	Categorical	Karachi, Hyderabad, Quetta, Larkana, Mirpurkhas, Dadu, Others	3964 (73.9), 416 (7.7), 122 (2.2), 59 (1.1), 59 (1.1), 53 (0.9), 690 (12.8)	

## Appendix B. Gelman Rubin Scores

**Table A3:** SBM Gelman Rubin score.

θ	$\hat{R}$	θ	$\hat{R}$	θ	$\hat{R}$
$\mu_{\beta }$	1.15	$\beta_{PCI\;timing}$	1.16	$\beta_{warfarin\;A}$	1.08
$\sigma_{\beta }$	1.15	$\beta_{CABG\;status}$	1.1	$\beta_{myocardial\;infarction}$	1.4
$\mu_{\beta_{0}}$	1.54	$\beta_{previous\;coronary\;bypass}$	1.2	$\beta_{BMI}$	1.08
$\sigma_{\beta_{0}}$	1.35	$\beta_{SustVTVF}$	1.09	$\beta_{intracar\;diacdevice}$	1.1
ϵ	2.06	$\beta_{Carotid\;disease}$	1.31	$\beta_{previous\;cv\;interventions}$	1.14
$\beta_{0}$	2.07	$\beta_{last\;wbc\;count}$	1.17	$\beta_{Statin\;A}$	1.08
$\beta_{Patient\;age}$	1.06	$\beta_{adp\;inhibitors\;within\;5days}$	1.13	$\beta_{ast\;hematocrit}$	1.31
$\beta_{Aortic\;Regurgitation}$	1.2	$\beta_{Arrhythmia}$	1.12	$\beta_{fhistory\;cad}$	1.14
$\beta_{last\;creatinine\;preop}$	1.05	$\beta_{Bronchodilators}$	1.23	$\beta_{Dyslipidemia}$	1.18
$\beta_{Tricuspid\;regurgitation}$	1.24	$\beta_{ejection\;fraction}$	1.06	$\beta_{steroids}$	1.05
$\beta_{pulmonary\;insufficiency}$	1.22	$\beta_{Diabetes}$	1.12	$\beta_{diastolic}$	1.18
$\beta_{Angina\;class}$	1.25	$\beta_{FirstCVSurgery}$	1.62	$\beta_{pulmonary\;artery\;hyper}$	1.14
$\beta_{AFibFlutter}$	1.21	$\beta_{Resuscitation}$	1.07	$\beta_{NYHA\;class}$	1.25
$\beta_{BPsystolic}$	1.12	$\beta_{MI\;timing}$	1.18	$\beta_{chronic\;lung\;disease}$	1.15
$\beta_{dialysis}$	1.11	$\beta_{anti\;coagulants}$	1.46	$\beta_{nitratesIV}$	1.26

**Table A4:** HBM Gelman Rubin score (level 0).

θ	$\hat{R}$	θ	$\hat{R}$	θ	$\hat{R}$
$\mu_{\beta }$	1.01	$\beta_{family\_history\_of\_coronary\_artery\_disease}$	1.0	$\beta_{Arrhythmia\;Type\;Sust\;VT/VF}$	1.0
$\sigma_{\beta }$	1.0	$\beta_{beta\_blockers}$	1.0	$\beta_{myocardial\_infarction}$	1.0
$\mu_{\beta_{0}}$	1.0	$\beta_{arrhythmia}$	1.0	$\beta_{resuscitation}$	1.0
$\sigma_{\beta_{0}}$	1.01	$\beta_{lipid\_lowering}$	1.0	$\beta_{dialysis}$	1.01
ϵ	1.1	$\beta_{Prior\_PCI}$	1.01	$\beta_{steroids}$	1.0
$\beta_{last\_cretenine\_preop}$	1.0	$\beta_{Cerebovascular\_disease}$	1.0	$\beta_{previous\_coronary\_bypass}$	1.0
$\beta_{last\_wbc\_count}$	1.0	$\beta_{Statin}$	1.0	$\beta_{previous\_valve}$	1.01
$\beta_{patient\_age}$	1.01	$\beta_{MI\_timing}$	1.01	$\beta_{FirstCVSurgery}$	1.0
$\beta_{last\_hematocrit}$	1.0	$\beta_{inotropes}$	1.01	$\beta_{warfarin}$	1.01
$\beta_{diastolic}$	1.01	$\beta_{Angina\_class}$	1.0	$\beta_{ace\_inhibitors}$	1.0
$\beta_{pulmonary\_insuff}$	1.01	$\beta_{PCI\_timing}$	1.0	$\beta_{Carotid\_disease}$	1.01
$\beta_{Mitral\_regurgitation}$	1.01	$\beta_{congestive\_heart\_failure}$	1.0	$\beta_{aspirin}$	1.0
$\beta_{Cardiac\_Presentation\_on\_Admission}$	1.0	$\beta_{adp\_inhibitors\_within\_5days}$	1.01	$\beta_{left\_main\_disease}$	1.01
$\beta_{gender\_id}$	1.0	$\beta_{cardiogenic\_shock}$	1.01	$\beta_{bronchodilators}$	1.0
$\beta_{CABG\_status}$	1.0	$\beta_{intracardiac\_device}$	1.0	$\beta_{Coronaries\_diseased}$	1.01
$\beta_{nitratesIV}$	1.0	$\beta_{pulmonary\_artery\_hypertension}$	1.0	$\beta_{BPsystolic}$	1.01
		$\beta_{NYHA\_class}$	1.01		

**Table A5:** HBM Gelman Rubin score (level 1).

θ	$\hat{R}$	θ	$\hat{R}$	θ	$\hat{R}$
$\beta_{\;last\_cretenine\_preop}$	1.0	$\beta_{\;Cerebovascular\_disease}$	1.0	$\beta_{\;previous\_coronary\_bypass}$	1.0
$\beta_{last\_wbc\_count}$	1.0	$\beta_{\;Statin}$	1.0	$\beta_{\;previou\_valve}$	1.01
${\beta \;}_{patient\_age}$	1.01	$\beta_{\;MI\_timing}$	1.01	$\beta_{\;FirstCVSurgery}$	1.0
$\beta_{\;last\_hematocrit}$	1.04	$\beta_{\;inotropes}$	1.01	$\beta_{\;warfarin}$	1.01
$\beta_{\;diastolic}$	1.01	$\beta_{\;Angina\_class}$	1.0	$\beta_{\;ace\_inhibitors}$	1.0
$\beta_{\;pulmonary\_insuff}$	1.01	$\beta_{\;PCI\_timing}$	1.0	$\beta_{\;Carotid\_disease}$	1.01
$\beta_{\;Mitral\_regurgitation}$	1.01	$\beta_{\;congestive\_heart\_failure}$	1.0	$\beta_{\;aspirin}$	1.0
$\beta_{\;Cardiac\_Presentation\_on\_Admission}$	1.04	$\beta_{\;adp\_inhibitors\_within\_5days}$	1.01	$\beta_{\;left\_main\_disease}$	1.01
$\beta_{\;gender\_id}$	1.01	$\beta_{\;cardiogenic\_shock}$	1.01	$\beta_{\;bronchodilators}$	1.03
$\beta_{\;CABG\_status}$	1.04	$\beta_{\;intracardiac\_device}$	1.04	$\beta_{\;Coronaries\_diseased}$	1.01
$\beta_{\;nitratesIV}$	1.01	$\beta_{\;pulmonary\_artery\_hypertension}$	1.0	$\beta_{BPsystolic}$	1.01
$\beta_{family\_history\_of\_coronary\_artery\_disease}$	1.0	$\beta_{\;NYHA\_class}$	1.01		
$\beta_{\;beta\_blockers}$	1.0	$\beta_{Arrhythmia\;Type\;Sust\;VT\;/\;VF}$	1.0		
$\beta_{\;arrhythmia}$	1.0	$\beta_{\;myocardial\_infarction}$	1.0		
$\beta_{\;lipid\_lowering}$	1.0	$\beta_{\;resuscitation}$	1.0		
$\beta_{\;Prior\_PCI}$	1.01	$\beta_{\;dialysis}$	1.01		
		$\beta_{\;steroids}$	1.0		

**Table A6:** HBM Gelman Rubin score (level 2).

θ	$\hat{R}$	θ	$\hat{R}$	θ	$\hat{R}$
$\beta_{\;last\_cretenine\_preop}$	1.0	$\beta_{\;Cerebovascular\_disease}$	1.0	$\beta_{\;previous\_coronary\_bypass}$	1.0
$\beta_{last\_wbc\_count}$	1.0	$\beta_{\;Statin}$	1.04	$\beta_{\;previou\_valve}$	1.01
${\beta \;}_{patient\_age}$	1.01	$\beta_{\;MI\_timing}$	1.01	$\beta_{\;FirstCVSurgery}$	1.0
$\beta_{\;last\_hematocrit}$	1.04	$\beta_{\;inotropes}$	1.01	$\beta_{\;warfarin}$	1.01
$\beta_{\;diastolic}$	1.01	$\beta_{\;Angina\_class}$	1.0	$\beta_{\;ace\_inhibitors}$	1.0
$\beta_{\;pulmonary\_insuff}$	1.01	$\beta_{\;PCI\_timing}$	1.0	$\beta_{\;Carotid\_disease}$	1.01
$\beta_{\;Mitral\_regurgitation}$	1.01	$\beta_{\;congestive\_heart\_failure}$	1.02	$\beta_{\;aspirin}$	1.0
$\beta_{\;Cardiac\_Presentation\_on\_Admission}$	1.04	$\beta_{\;adp\_inhibitors\_within\_5days}$	1.01	$\beta_{\;left\_main\_disease}$	1.01
$\beta_{\;gender\_id}$	1.01	$\beta_{\;cardiogenic\_shock}$	1.01	$\beta_{\;bronchodilators}$	1.03
$\beta_{\;CABG\_status}$	1.04	$\beta_{\;intracardiac\_device}$	1.04	$\beta_{\;Coronaries\_diseased}$	1.01
$\beta_{\;nitratesIV}$	1.01	$\beta_{\;pulmonary\_artery\_hypertension}$	1.0	$\beta_{BPsystolic}$	1.01
$\beta_{family\_history\_of\_coronary\_artery\_disease}$	1.0	$\beta_{\;NYHA\_class}$	1.01		
$\beta_{\;beta\_blockers}$	1.0	$\beta_{Arrhythmia\;Type\;Sust\;VT\;/\;VF}$	1.0		
$\beta_{\;arrhythmia}$	1.0	$\beta_{\;myocardial\_infarction}$	1.0		
$\beta_{\;lipid\_lowering}$	1.0	$\beta_{\;resuscitation}$	1.0		
$\beta_{\;Prior\_PCI}$	1.01	$\beta_{\;dialysis}$	1.01		
		$\beta_{\;steroids}$	1.0		

**Table A7:** HBM Gelman Rubin score (level 3).

θ	$\hat{R}$	θ	$\hat{R}$	θ	$\hat{R}$
$\beta_{\;last\_cretenine\_preop}$	1.0	$\beta_{\;Cerebovascular\_disease}$	1.0	$\beta_{\;previous\_coronary\_bypass}$	1.0
$\beta_{last\_wbc\_count}$	1.0	$\beta_{\;Statin}$	1.0	$\beta_{\;previou\_valve}$	1.01
${\beta \;}_{patient\_age}$	1.01	$\beta_{\;MI\_timing}$	1.01	$\beta_{\;FirstCVSurgery}$	1.0
$\beta_{\;last\_hematocrit}$	1.04	$\beta_{\;inotropes}$	1.01	$\beta_{\;warfarin}$	1.01
$\beta_{\;diastolic}$	1.01	$\beta_{\;Angina\_class}$	1.04	$\beta_{\;ace\_inhibitors}$	1.0
$\beta_{\;pulmonary\_insuff}$	1.01	$\beta_{\;PCI\_timing}$	1.01	$\beta_{\;Carotid\_disease}$	1.01
$\beta_{\;Mitral\_regurgitation}$	1.01	$\beta_{\;congestive\_heart\_failure}$	1.0	$\beta_{\;aspirin}$	1.0
$-\beta_{\;Cardiac\_Presentation\_on\_Admission}$	1.0	$\beta_{\;adp\_inhibitors\_within\_5days}$	1.01	$\beta_{\;left\_main\_disease}$	1.01
$\beta_{\;gender\_id}$	1.0	$\beta_{\;cardiogenic\_shock}$	1.01	$\beta_{\;bronchodilators}$	1.03
$\beta_{\;CABG\_status}$	1.04	$\beta_{\;intracardiac\_device}$	1.04	$\beta_{\;Coronaries\_diseased}$	1.01
$\beta_{\;nitratesIV}$	1.01	$\beta_{\;pulmonary\_artery\_hypertension}$	1.0	$\beta_{BPsystolic}$	1.01
$\beta_{family\_history\_of\_coronary\_artery\_disease}$	1.0	$\beta_{\;NYHA\_class}$	1.01		
$\beta_{\;beta\_blockers}$	1.0	$\beta_{Arrhythmia\;Type\;Sust\;VT\;/\;VF}$	1.0		
$\beta_{\;arrhythmia}$	1.0	$\beta_{\;myocardial\_infarction}$	1.0		
$\beta_{\;lipid\_lowering}$	1.0	$\beta_{\;resuscitation}$	1.0		
$\beta_{\;Prior\_PCI}$	1.01	$\beta_{\;dialysis}$	1.01		
		$\beta_{\;steroids}$	1.0		

## Appendix C. Estimated Coefficient Values & Feature Importance

**Table A8:** SBM estimated coefficient values.

Variable	θ	±sd	Variable	θ	±sd	Variable	θ	±sd
Patient age	0.086	±0.036	Carotid disease	0.099	±0.041	BMI	0.04	±0.054
Aortic Regurgitation	0.125	±0.04	last wbc count	0.01	±0.028	intracardiac device	0.061	±0.051
last creatinine preop	0.146	±0.048	adp inhibitors within 5 days	0.044	±0.046	previous cv interventions	0.078	±0.053
Tricuspid regurgitation	0.084	±0.036	Arrhythmia	0.065	±0.055	Statin A	0.062	±0.061
pulmonary insufficiency	0.118	±0.057	Bronchodilators	0.094	±0.05	last hematocrit	0.075	±0.055
Angina class	0.074	±0.054	ejection fraction	0.096	±0.057	f history cad	0.08	±0.059
AFibFlutter	0.119	±0.043	Diabetes	0.09	±0.069	Dyslipidemia	0.055	±0.055
BPsystolic	0.034	±0.025	FirstCVSurgery	0.084	±0.07	steroids	0.057	±0.064
dialysis	0.083	±0.048	Resuscitation	−0.03	±0.051	diastolic	0.052	±0.058
left main disease	0.107	±0.057	MI timing	0.087	±0.024	pulmonary artery hyper	0.054	±0.062
PCI timing	0.069	±0.031	anti coagulants	0.073	±0.052	NYHA class	0.094	±0.063
CABG status	0.032	±0.039	previous valve	0.056	±0.066	chronic lung disease	0.103	±0.065
previous coronary bypass	−0.014	±0.035	congestive heart failure A	0.079	±0.044	nitratesIV	0.059	±0.066
SustVTVF	0.112	±0.061	warfarin A	0.12	±0.033	cardiogenic shock	0.085	±0.068
			myocardial infarction	0.079	±0.055	Coronaries diseased	0.034	±0.048

**Table A9:** MLR estimated coefficient values.

Variable	θ	Variable	θ	Variable	θ
last_cretenine_preop	0.293081	arrhythmia	1.646449	SustVTVF	−1.24274
last_wbc_count	0.171635	lipid_lowering_A	0.143871	myocardial_infarction	−0.22586
patient_age	0.223315	Prior_PCI	−2.74268	resuscitation	0.454801
BPsystolic	0.135273	Cerebovascular_disease	0.275619	dialysis	−0.46064
last_hematocrit	−0.12409	Statin_A	0.065754	steroids	2.651557
diastolic	−0.11174	MI_timing	0.114422	previous_coronary_bypass	−2.2 × 10^−16^
pulmonary_insuff	0.10158	inotropes	−1.11074	previous_valve	2.22 × 10^−16^
Mitral_regurgitation	0.087977	Angina_class	0.026034	FirstCVSurgery	8.88 × 10^−16^
Cardiac_Presentation_on_Admission	0.020097	PCI_timing	−1.36871	warfarin_A	−2.2 × 10^−16^
gender_id	0.32052	congestive_heart_failure_A	0.191495	ace_inhibitors_A	−0.23953
CABG_status	0.347848	adp_inhibitors_within_5days	0.361978	Carotid_disease	−0.33631
nitratesIV	0.31499	cardiogenic_shock	0.400921	aspirin_A	−0.2182
f_history_cad	0.12583	intracardiac_device	0.172755	left_main_disease	−0.09115
beta_blockers_A	−0.32623	pulmonary_artery_hypertension	2.171717	bronchodilators	−0.4348
		NYHA_class	0.123221	Coronaries_diseased	0.155315

**Table A10:** SVR estimated coefficient values.

Variable	θ	Variable	θ	Variable	θ
last_cretenine_preop	0.300778	arrhythmia	0.515685	SustVTVF	0.172685
last_wbc_count	0.046161	lipid_lowering_A	0.0337	myocardial_infarction	−0.0819
patient_age	0.085908	Prior_PCI	−0.30512	resuscitation	2
BPsystolic	0.040163	Cerebovascular_disease	0.109956	dialysis	0.160044
last_hematocrit	−0.00757	Statin_A	0.05522	steroids	0
diastolic	−0.0208	MI_timing	0.019536	previous_coronary_bypass	0
pulmonary_insuff	−8.6× 10^−5^	inotropes	−0.67392	previous_valve	0
Mitral_regurgitation	0.020046	Angina_class	0.022927	FirstCVSurgery	0
Cardiac_Presentation_on_Admission	8.61× 10^−5^	PCI_timing	−0.14735	warfarin_A	0
gender_id	0.31685	congestive_heart_failure_A	0.440434	ace_inhibitors_A	−0.1476
CABG_status	0.119829	adp_inhibitors_within_5days	0.076298	Carotid_disease	0.09922
nitratesIV	0.161362	cardiogenic_shock	0.425116	aspirin_A	−0.05569
f_history_cad	0.017713	intracardiac_device	−0.14727	left_main_disease	−0.03404
beta_blockers_A	−0.12152	pulmonary_artery_hypertension	1.672917	bronchodilators	−0.14199
		NYHA_class	0.038393	Coronaries_diseased	0.077312

**Table A11:** Lasso estimated coefficient values.

Variable	θ	Variable	θ	Variable	θ
last_cretenine_preop	0.24022	arrhythmia	0.565138	SustVTVF	0
last_wbc_count	0.167671	lipid_lowering_A	0	myocardial_infarction	0
patient_age	0.231557	Prior_PCI	0	resuscitation	0
BPsystolic	0.107887	Cerebovascular_disease	0.07128	dialysis	0
last_hematocrit	−0.12907	Statin_A	0	steroids	0
diastolic	−0.08049	MI_timing	0.069182	previous_coronary_bypass	0
pulmonary_insuff	0.082534	inotropes	0	previous_valve	0
Mitral_regurgitation	0.098205	Angina_class	0.030879	FirstCVSurgery	0
Cardiac_Presentation_on_Admission	0.000971	PCI_timing	0	warfarin_A	0
gender_id	0.265267	congestive_heart_failure_A	0.07735	ace_inhibitors_A	−0.17409
CABG_status	0.275316	adp_inhibitors_within_5days	0.222577	Carotid_disease	0
nitratesIV	0.228018	cardiogenic_shock	0	aspirin_A	−0.06411
f_history_cad	0.075935	intracardiac_device	0	left_main_disease	−0.02798
beta_blockers_A	−0.19498	pulmonary_artery_hypertension	0.391082	bronchodilators	0
		NYHA_class	0.133256	Coronaries_diseased	0.05406

**Table A12:** Ridge estimated coefficient values.

Variable	θ	Variable	θ	Variable	θ
last_cretenine_preop	0.29287	arrhythmia	1.63379	SustVTVF	−1.22867
last_wbc_count	0.17179	lipid_lowering_A	0.14247	myocardial_infarction	−0.22742
patient_age	0.22354	Prior_PCI	−2.16652	resuscitation	0.44811
BPsystolic	0.13518	Cerebovascular_disease	0.27530	dialysis	−0.45755
last_hematocrit	−0.12417	Statin_A	0.06717	steroids	2.39899
diastolic	−0.11162	MI_timing	0.11477	previous_coronary_bypass	0.00000
pulmonary_insuff	0.10174	inotropes	−1.09653	previous_valve	0.00000
Mitral_regurgitation	0.08837	Angina_class	0.02591	FirstCVSurgery	0.00000
Cardiac_Presentation_on_Admission	0.02033	PCI_timing	−1.08030	warfarin_A	0.00000
gender_id	0.32028	congestive_heart_failure_A	0.19182	ace_inhibitors_A	−0.23946
CABG_status	0.34619	adp_inhibitors_within_5days	0.36213	Carotid_disease	−0.32121
nitratesIV	0.31625	cardiogenic_shock	0.38599	aspirin_A	−0.21897
f_history_cad	0.12597	intracardiac_device	0.16868	left_main_disease	−0.09149
beta_blockers_A	−0.32527	pulmonary_artery_hypertension	2.15283	bronchodilators	−0.43283
		NYHA_class	0.12320	Coronaries_diseased	0.15501

**Table A13:** HR estimated coefficient values.

Variable	θ	Variable	θ	Variable	θ
last_cretenine_preop	0.27735	arrhythmia	0.58070	SustVTVF	−0.01454
last_wbc_count	0.05527	lipid_lowering_A	0.08576	myocardial_infarction	−0.07164
patient_age	0.13353	Prior_PCI	−1.22463	resuscitation	3.02020
BPsystolic	0.03054	Cerebovascular_disease	0.14033	dialysis	0.21827
last_hematocrit	−0.01369	Statin_A	0.04562	steroids	0.08374
diastolic	−0.00455	MI_timing	0.01957	previous_coronary_bypass	0.00000
pulmonary_insuff	0.00650	inotropes	−1.03363	previous_valve	0.00000
Mitral_regurgitation	0.03187	Angina_class	0.03338	FirstCVSurgery	0.00000
Cardiac_Presentation_on_Admission	0.00038	PCI_timing	−0.61239	warfarin_A	0.00000
gender_id	0.25862	congestive_heart_failure_A	0.24248	ace_inhibitors_A	−0.18392
CABG_status	0.14018	adp_inhibitors_within_5days	0.10193	Carotid_disease	0.19897
nitratesIV	0.09323	cardiogenic_shock	0.62368	aspirin_A	−0.06419
f_history_cad	0.02320	intracardiac_device	−0.20054	left_main_disease	−0.03968
beta_blockers_A	−0.15233	pulmonary_artery_hypertension	1.65099	bronchodilators	−0.18209
		NYHA_class	0.04937	Coronaries_diseased	0.12048

**Table A14:** SGD estimated coefficient values.

Variable	θ	Variable	θ	Variable	θ
last_cretenine_preop	0.30488	arrhythmia	0.79300	SustVTVF	−0.05269
last_wbc_count	0.14100	lipid_lowering_A	0.11070	myocardial_infarction	−0.17027
patient_age	0.27325	Prior_PCI	1.99228	resuscitation	−0.00445
BPsystolic	0.18533	Cerebovascular_disease	0.24745	dialysis	−0.30446
last_hematocrit	−0.12108	Statin_A	0.10900	steroids	0.07558
diastolic	−0.05952	MI_timing	0.14651	previous_coronary_bypass	0.00000
pulmonary_insuff	0.12881	inotropes	−0.34767	previous_valve	0.00000
Mitral_regurgitation	0.08367	Angina_class	0.03855	FirstCVSurgery	0.00000
Cardiac_Presentation_on_Admission	−0.00783	PCI_timing	1.04908	warfarin_A	0.00000
gender_id	0.35086	congestive_heart_failure_A	0.19975	ace_inhibitors_A	−0.22757
CABG_status	0.33625	adp_inhibitors_within_5days	0.35751	Carotid_disease	0.00765
nitratesIV	0.31133	cardiogenic_shock	−0.13044	aspirin_A	−0.16028
f_history_cad	0.14661	intracardiac_device	0.01240	left_main_disease	−0.08449
beta_blockers_A	−0.26874	pulmonary_artery_hypertension	0.61975	bronchodilators	−0.21460
		NYHA_class	0.14968	Coronaries_diseased	0.28836
